# Supplementary material for: SCISSOR: a framework for identifying structural changes in RNA transcripts
Source: Nat Commun. 2021 Jan 12;12:286. doi: 10.1038/s41467-020-20593-3 (PMC7804101; doi:10.1038/s41467-020-20593-3)
Supplement: Supplementary file 1 — Supplementary Information [file 41467_2020_20593_MOESM1_ESM.pdf]

Supplementary Information

Contents

Supplementary Tables **2**

Supplementary Figures **5**

Supplementary Note 1: Normalization **21**

Supplementary Note 2: *TP53* splice site mutations in HNSC **25**

Supplementary Note 3: Methods comparison **28**

Supplementary Note 4: Gene filtering method **37**

Supplementary Note 5: Modified projection outlyingness **39**

Supplementary Note 6:
Non-recurrent events in infrequently mutated genes **41**

Supplementary Note 7: Evaluation of small deletion events **41**

Supplementary Note 8:
Evaluation of common structure of RNA-seq samples **44**

Supplementary Tables

(a) Global shape changes

Outlier	Statistic	Class	Region	Region.tag	VJF	Type
164	57.95	MES	629∼3304	J13	0.948	Global
69	45.65	IR	630∼1033	I3	0.677	Global
16	34.05	IR	1412∼1815	I5	0.323	Global
185	33.29	ATT	1∼1033	ATT3	0.667	Global
216	31.72	IR	1926∼2268	I6	0.58	Global
119	25.64	IR	2572∼2766	I8	0.704	Global
416	22.76	IR	1926∼2268	I6	0.2	Global

(b) Local shape changes

Outlier	Statistic	Class	Region	Region.tag	VJF	Type
110	13.57	Cryptic_ES	2498∼2516	E8	0.414	Local
112	12.55	Del	2272∼2321	E7		Local
114	12.46	Del	1112∼1161	E4		Local
356	10.81	Del	1172∼1217	E4		Local
286	10.72	Del	1172∼1217	E4		Local
239	6.84	Del	1034∼1054	E4		Local
63	6.59	Cryptic_IR	32603̃304	I9	0.106	Local

Supplementary Table 1: The *TP53* results tables from SCISSOR. The first few rows of the results tables from (a) the global shape change detection and (b) local shape change detection at the gene *TP53*. The results of SCISSOR at a single gene are reported as a table summarizing the information about the identified outliers. Each table includes the case IDs of the identified outliers, the corresponding statistics, class of outliers, regions where shape changes detected, region.tag (tags for the high-dimensional direction where the modified projection outlyingness was maximized), variant junction frequency (VJF). VJF is available when the event is involved with splicing aberration. ES: exon skipping, MES: multiple exon skipping, IR: intron retention, ATT: alternative transcript termination, Del: deletion.

Sample barcode	Splice site mutation (VAF)	Splice site	Other mutations	RSEM (percentile)	A	B1	B2	Ratio
CN-5370	g.chr17:7578555C>T (0.31)	intronic	Missense	1468.6 (57.3)	241	-	101	0.42
CQ-5326	g.chr17:7579698A>C (0.17)	intronic	Missense	637.8 (26.8)	49	-	16	0.33
CV-7432	g.chr17:7579312C>A (0.3)	exonic (LBE)	Frame shift del	136.2 (1.1)	10	3	-	0.30
CN-6012	g.chr17:7578553T>G (0.44)	exonic (LBE-1)	-	2594.9 (85.2)	257	-	51	0.20
CN-4740	g.chr17:7579591C>T (0.09)	intronic	Nonsense	850.5 (33.8)	48	-	8	0.17
CQ-5331	g.chr17:7576927C>G (0.18)	exonic	Missense	1278.6 (51.3)	215	-	11	0.05
UF-A71A	g.chr17:7578176C>A (0.4)	intronic	Missense	616.6 (25.7)	20	1	-	0.05
CV-5977	g.chr17:7576853C>T (0.31)	exonic (LBE)	Missense	1347.3 (53.8)	86	4	-	0.04
KU-A6H8	g.chr17:7579592T>A (0.03)	intronic	-	1108.5 (44.5)	26	-	1	0.03
QK-A6VB	g.chr17:7578370C>T (0.46)	intronic	Missense	2334.5 (81.2)	163	-	6	0.03
CR-7376	g.chr17:7579698_7579699insCC (0.15)	intronic	Missense	1403.5 (55.1)	108	-	-	0
CN-4726	g.chr17:7579310A>T (0.41)	intronic	-	229.6 (6.6)	10	-	-	0
CV-7430	g.chr17:7578553T>C (0.65)	exonic (LBE-1)	-	2261.4 (79.4)	211	-	-	0
D6-6825	g.chr17:7578369A>C (0.44)	intronic	-	331.9 (12.6)	17	-	-	0
CR-7401	g.chr17:7578177C>T (0.14)	exonic (LBE)	-	760.8 (30.8)	35	-	-	0
CN-6020	g.chr17:7578177C>A (0.57)	exonic (LBE)	-	253.2 (7.7)	13	-	-	0

Supplementary Table 2: The *TP53* splice site mutations for the 16 samples that were not identified by SCISSOR. For each sample in a row, the TCGA barcode (Column 1), information of the splice site mutation (Column 2 & 3), other mutations if any (Column 4), the normalized RSEM value (Column 5), and the numbers (Column 6-8; A=# junction reads, B1=# retained reads, B2=# skipped reads) used for computing the abnormal read ratio (Column 9) are provided. The rows are sorted by the ratio of abnormal reads. In the table, LBE represents the last base of exon (both 3' and 5') and LBE-1 indicates one base of LBE on the exonic side.

Pathway	% of genes	P-value	FDR
Cell-cell adhesion	9.2%	3×10^{-18}	6×10^{-15}
Cell adhesion	9.2%	6×10^{-11}	1×10^{-07}
Extracellular matrix organization	5.8%	2×10^{-10}	4×10^{-07}
Collagen catabolic process	3.2%	2×10^{-8}	4×10^{-05}
Response to cAMP	2.4%	1×10^{-6}	2×10^{-03}
Positive regulation of fibroblast proliferation	2.4%	4×10^{-6}	7×10^{-03}
Positive regulation of transcription from RNA polymerase II promoter	11.4%	6×10^{-6}	0.01
Cell proliferation	6.1%	6×10^{-6}	0.01
Extracellular matrix disassembly	2.7%	1×10^{-5}	0.02

Supplementary Table 3: Results from a gene ontology analysis (DAVID). The gene list where widely altered samples have shape changes was analyzed using DAVID to highlight the most relevant pathways associated with the genes in the list[1]. For each pathway, the percentage of genes in the pathway, an exact one-sided p-value, and false discovery rate (FDR) from DAVID are reported in the second and third columns, respectively.

Supplementary Figures

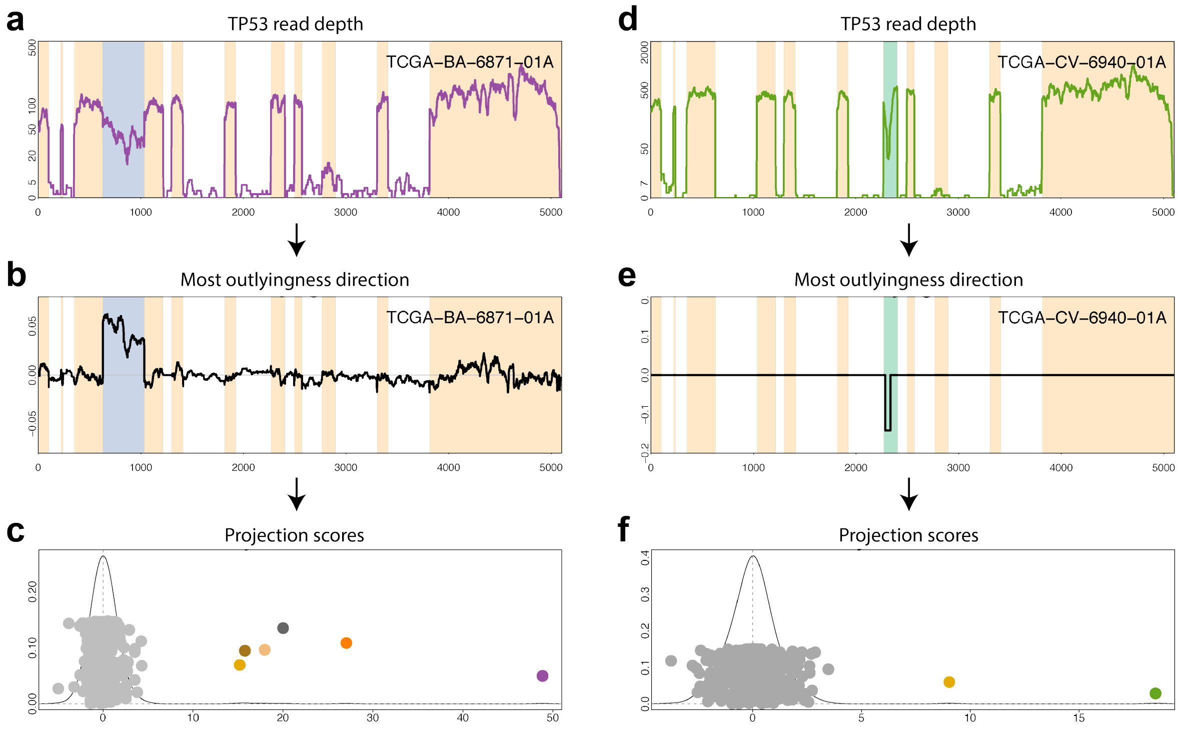

Supplementary Figure 1: Examples of global shape changes (GSCs), local shape changes (LSCs), and most outlying directions (MODs) from *TP53*. (a) An example of GSCs. An identified global shape change, (b) the corresponding MOD, and (c) the projection scores obtained by projecting all the normalized samples onto this MOD with a kernel density estimate are shown. This MOD characterizes the abnormality of the particular outlier by the gain of the 3rd intron as highlighted by the colored background. The distribution of the projection scores clearly separate several colored points, providing a group of samples that share the analogous abnormality. The samples in this group are shown in Group 2 of Supplementary Fig. 2 and Group 4 of Supplementary Fig. 4 using the same colors. (d) An example of LSCs. (e) The MOD of this outlier informs the local abnormality as highlighted by the green background. (f) The two samples are outlying in this direction as described in Group 4 of Supplementary Fig. 3 using the same colors.

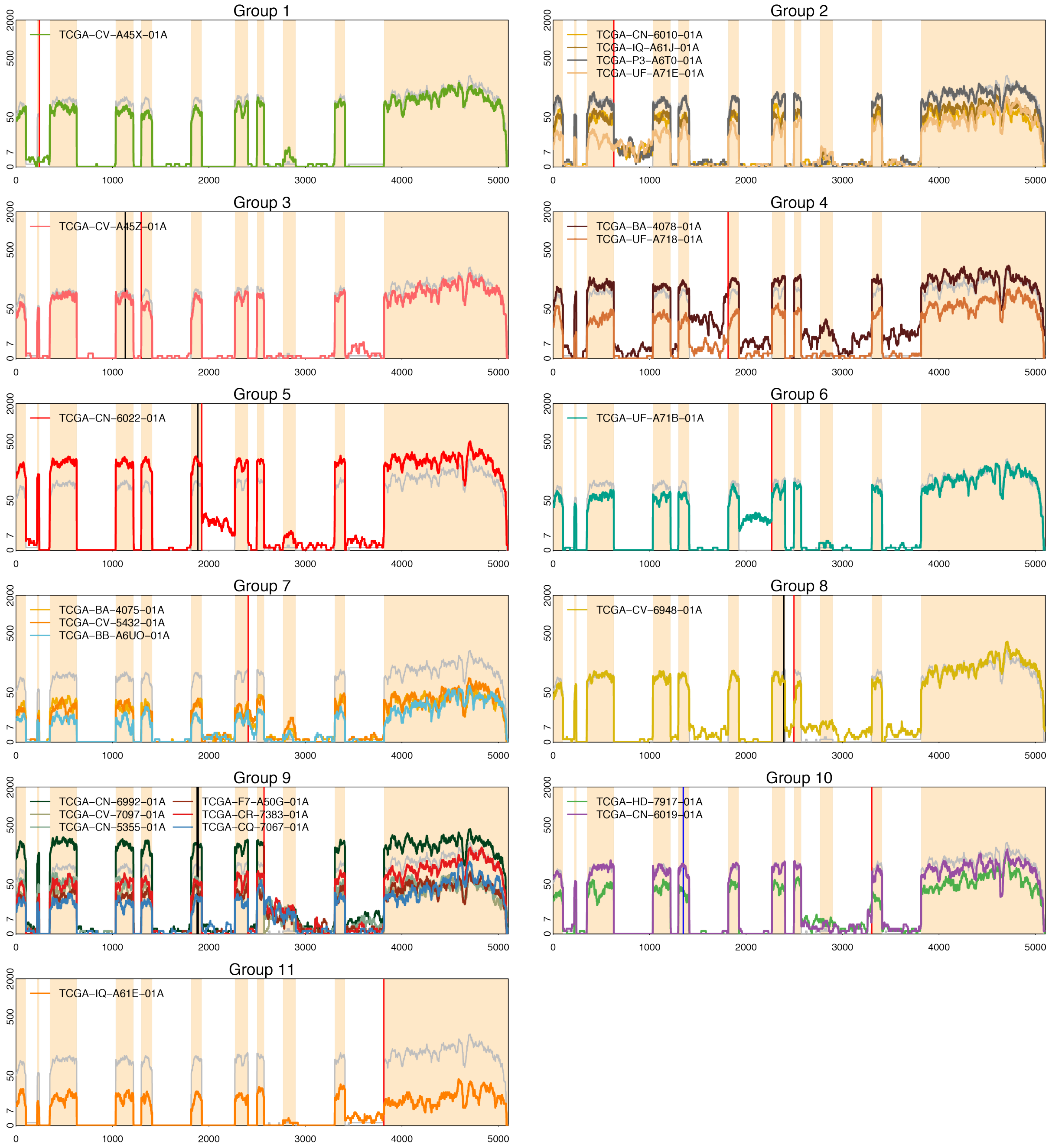

Supplementary Figure 2: Splice mutations identified by SCISSOR in *TP53*. (Caption next page.)

Supplementary Figure 2: Splice mutations identified by SCISSOR in *TP53*. The 23 shape outliers involved in splice site mutations are displayed. The outliers are grouped by the sites of the mutations indicated by red vertical lines and the outliers in each group exhibit similar splicing aberrations in a manner that would be expected near the splice site mutations. The groups are ordered by the positions of splice site mutations. A black vertical line indicates position of a mutation other than splice site mutations. Group 1 presents one sample with the small second exon skipped. Group 2 includes four samples that retains the third intron. Group 3 presents one sample showing the fifth exon fractionally skipped. Group 4 demonstrates two sample outliers that retain the fifth intron. Group 5 and 6 show two samples with the sixth intron retained although the positions of mutations are different in the two samples. Group 7 presents three samples with the seventh intron retained. Group 8 presents one sample demonstrating that a small fraction of the eighth exon are skipped. Group 9 includes six samples that retain the eighth intron. Group 10 shows two samples that retain the last small part of the ninth intron. Lastly, one sample in Group 11 has the last exon fractionally skipped.

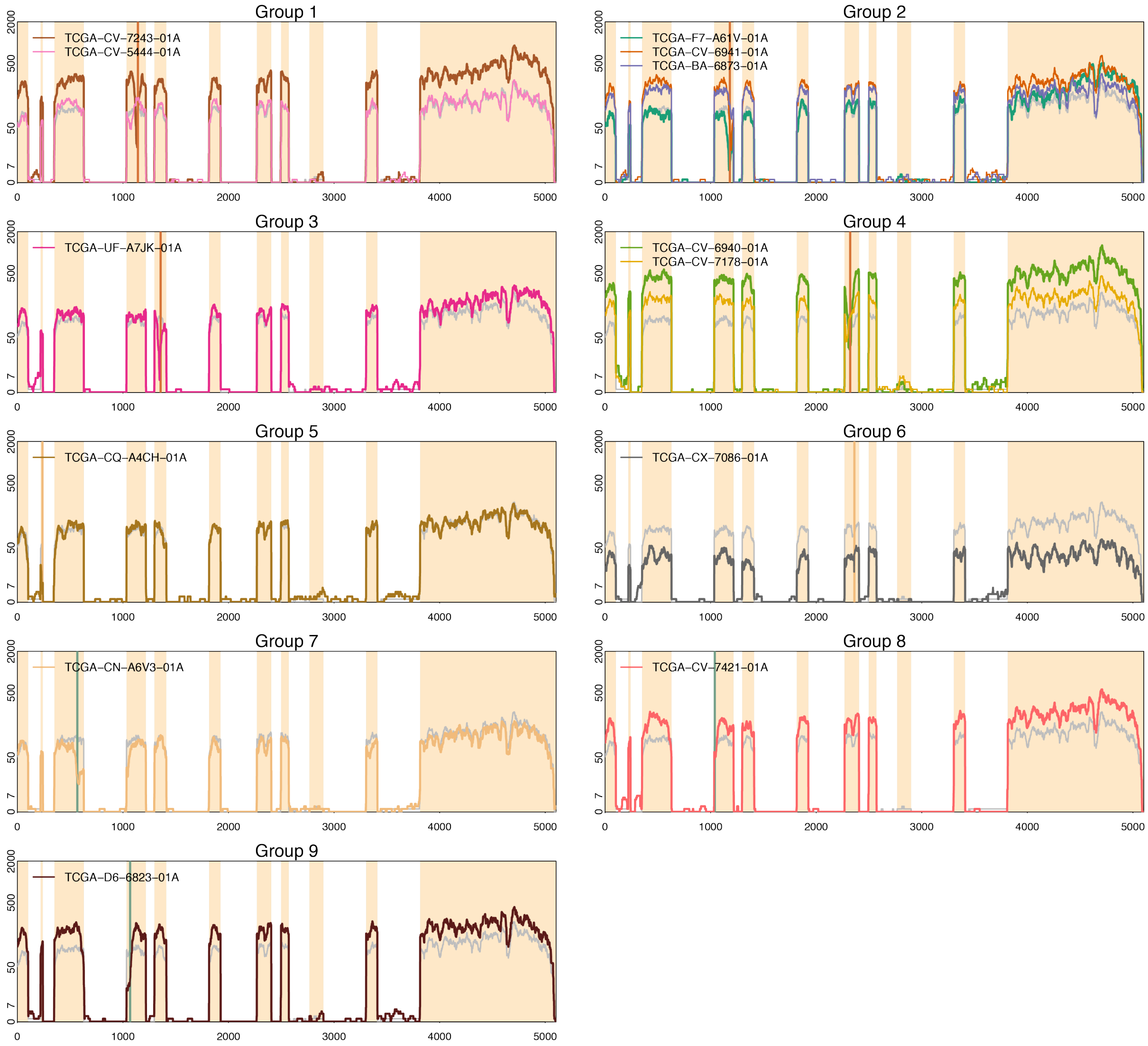

Supplementary Figure 3: Shape changes associated with mutations other than splice site in *TP53*. The shape changes (n=13) associated with mutations other than splice site mutations are classified into 9 groups. All these shape changes were identified by the local method and demonstrate small deletions near the mutations. In each plot, a vertical line indicates position of mutations. Group 1-4 have in-frame deletions indicated by the vertical lines in each plot. Group 5 & 6 have frameshift deletions. Group 7-9 have missense mutations.

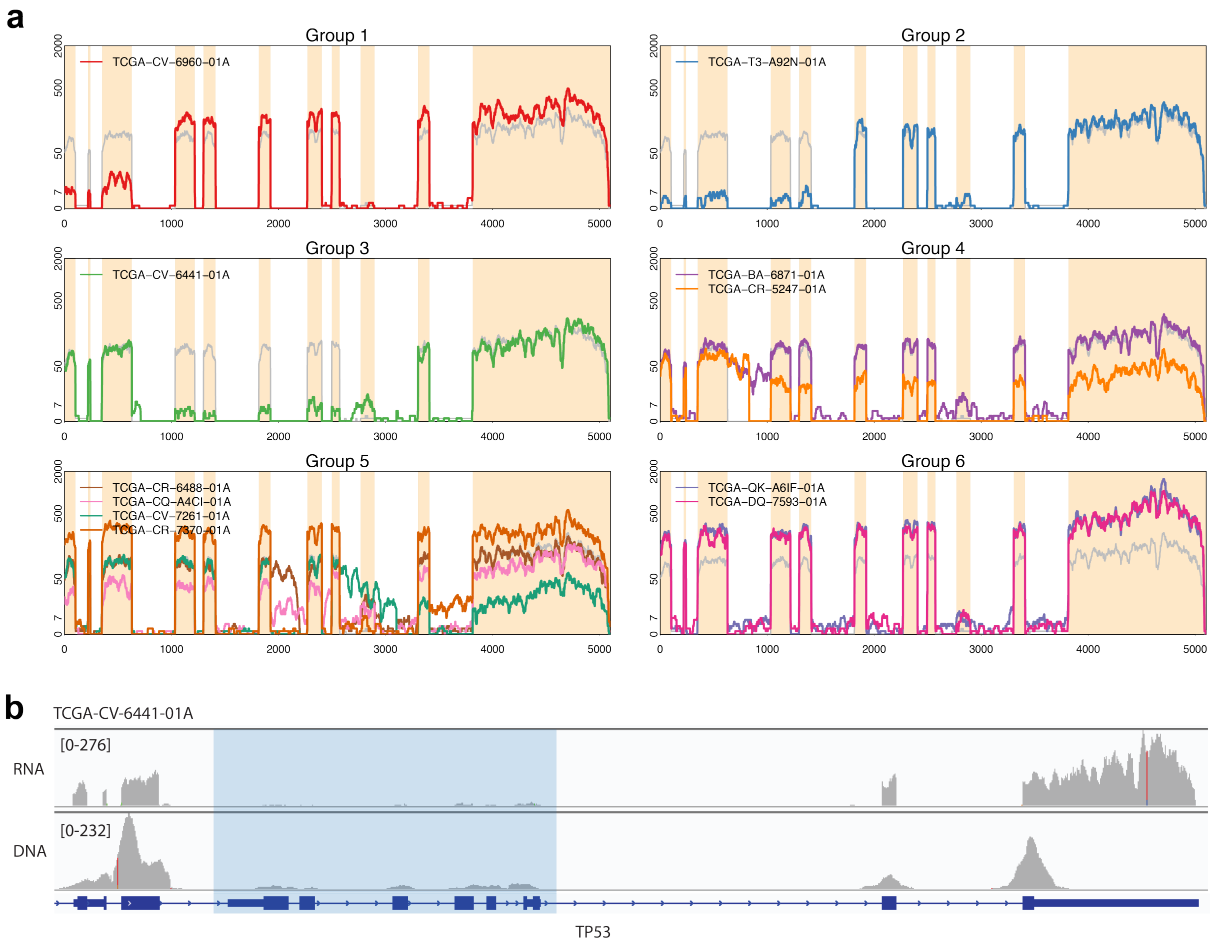

Supplementary Figure 4: Shape changes in the absence of mutations in *TP53*. (a) Shape outliers (n=11) with no directly correlated mutations are displayed. The outliers are grouped by the types of shape aberrations. The samples in Groups 1-3 exhibit distinct types of structural variants. In Group 1 and 2, the first few exons were deleted. In Group 3, exons 3-7 were deleted in the middle of the gene. The group 4 abnormally retains the 3rd intron and the group 5 retains several different intronic regions. The samples in group 6 are highly expressed and exhibit moderate levels of intron retention. (b) Reads distributions of the sample in Group 3 are shown for RNA-seq (top) and DNA-seq (bottom) using IGV. The exons skipped in RNA-seq profile are consistently deleted in DNA-seq, indicating intragenic deletions.

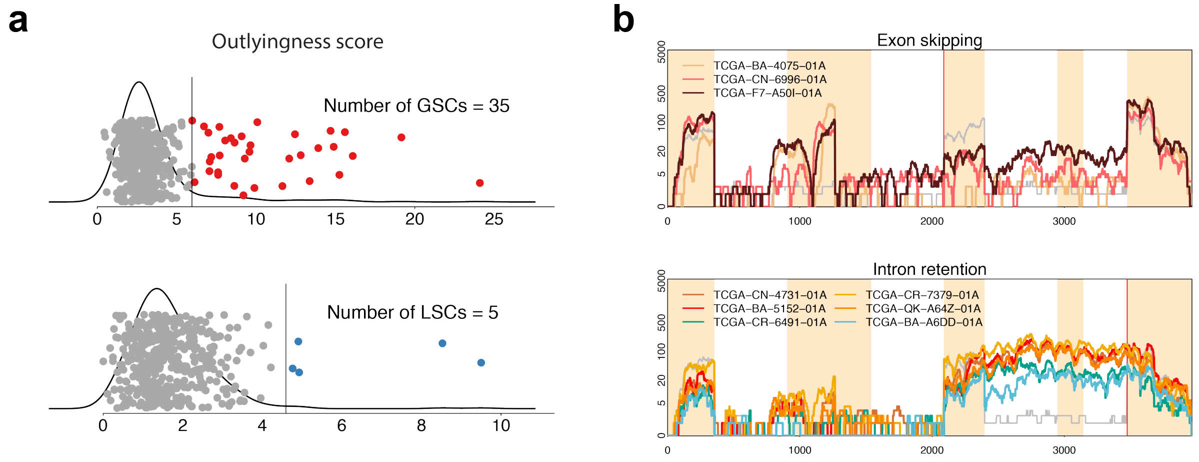

Supplementary Figure 5: Outlyingness statistics and shape changes driven by splice site mutations at *CDKN2A*. (a) In *CDKN2A*, the 35 and 5 shape changes were identified from the global method and local method, respectively. The nine splice site mutations called at *CDKN2A* were all identified and shown in the panel (b). (b) Top, three of the nine samples had splice site mutations at the 3' of the 2nd intron and skipped the following exon. Bottom, the other six samples had splice site mutations at the 3' of the 4th exon and retained the preceding intron.

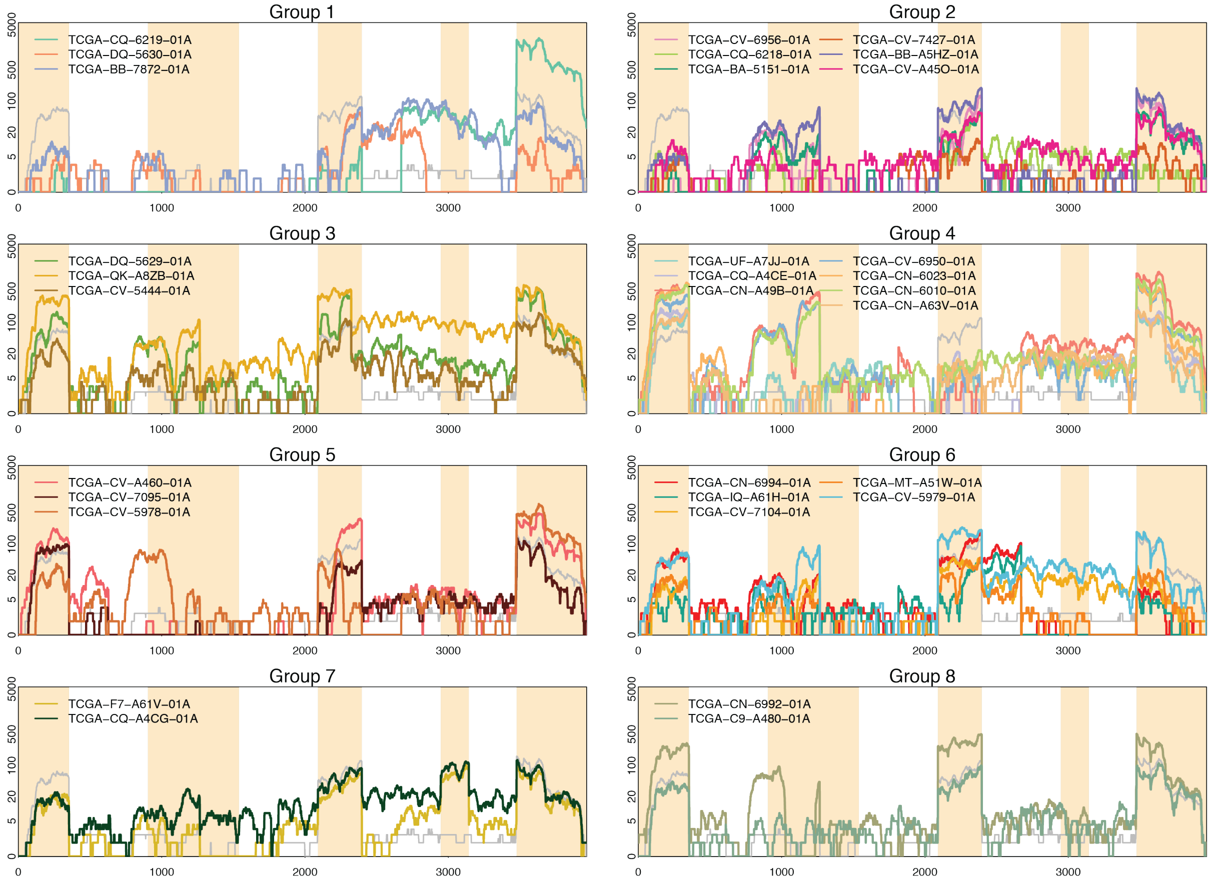

Supplementary Figure 6: Shape changes at *CDKN2A*. All the identified shape outliers without splice site mutations at *CDKN2A* are shown (n=31). The outliers were classified into 8 groups by their distinct shapes. Group 1 skips the first and a part of the second exon followed by usually high intron. Group 2 skips the first exon. Group 3-5 demonstrates aberrant splicing of the 3rd exon. Group 6 exhibits abnormal intronic expression with partial loss of the last exon. Group 7 exhibits a differential usage of the 4th exon. In Group 8, TCGA-CN-6992-01A shows a distinct pattern in the 2nd exon and TCGA-C9-A480-01A appears slightly high 3rd exon compared to the 1st and last exons although they are not apparent from the sample overlays.

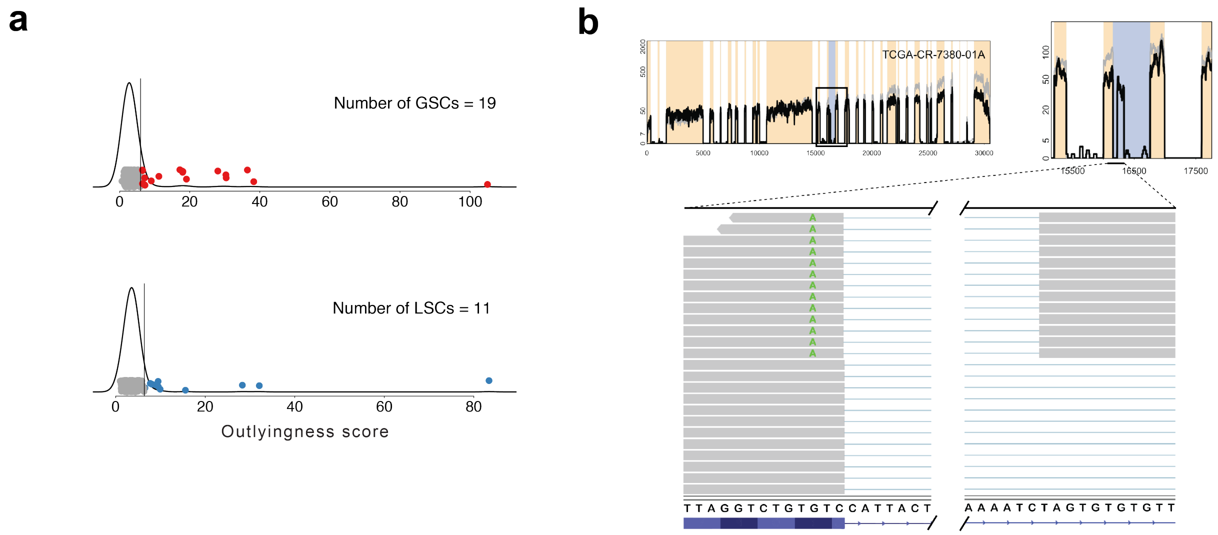

Supplementary Figure 7: Results from *FAT1*. (a) In *FAT1*, the 19 GSCs (global shape changes) and 11 LSCs (local shape changes) were identified. Interestingly, the outlyingness scores of the shape outliers are mostly far beyond the cutoff values, indicating that the shape changes involved in *FAT1* are likely to be drastic (Supplementary Fig. 8). (b) An example of the LSCs (left) is shown with a zoom-in figure on its local abnormality (right). We found that this outlier differentially used a novel exon in the colored region. More interestingly, we observed every junction read splicing into this novel exon has exonic mutation (Chr4:g.187,535,347G>A) . This strongly implicates that this exonic mutation might be responsible for the differential exon usage.

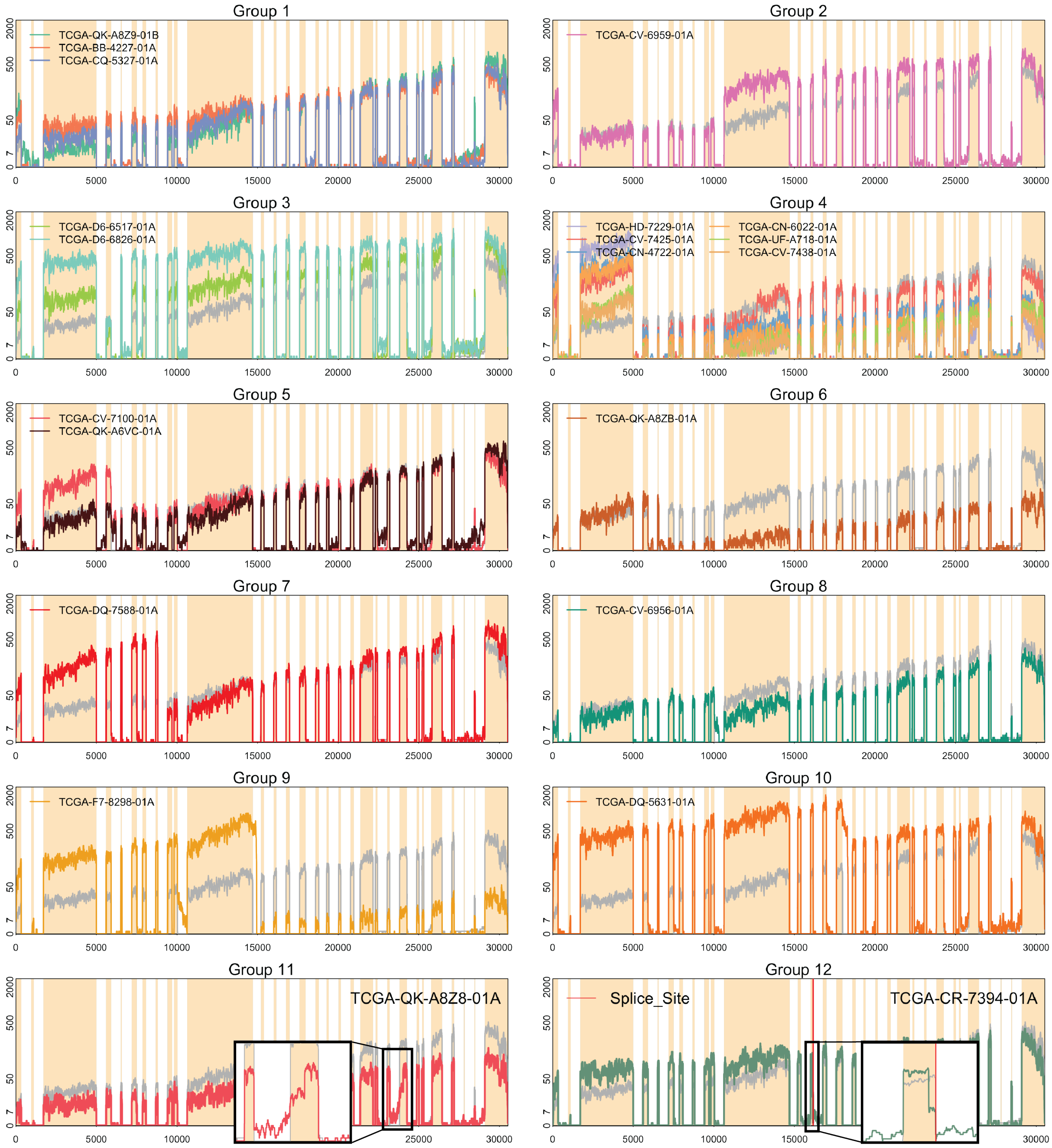

Supplementary Figure 8: Shape changes at *FAT1*. In *FAT1*, 30 samples were identified by SCISSOR demonstrating distinct shape changes in expression. Among them, the outliers associated with abnormal splicing are shown (n=21). These splicing outliers are manually classified into the 12 groups by their distinct shape change patterns. Group 1 presents the first exon with unusually high expression compared to the following exons. Group 2 includes one sample that skips several exons from the third to tenth. Group 3 abnormally skips the fourth exon. Outliers in Group 4-10 present structural variants that express only the front part of the gene and skip the rest of the gene, indicating fusion events. Group 11 and 12 present cryptic splice sites.

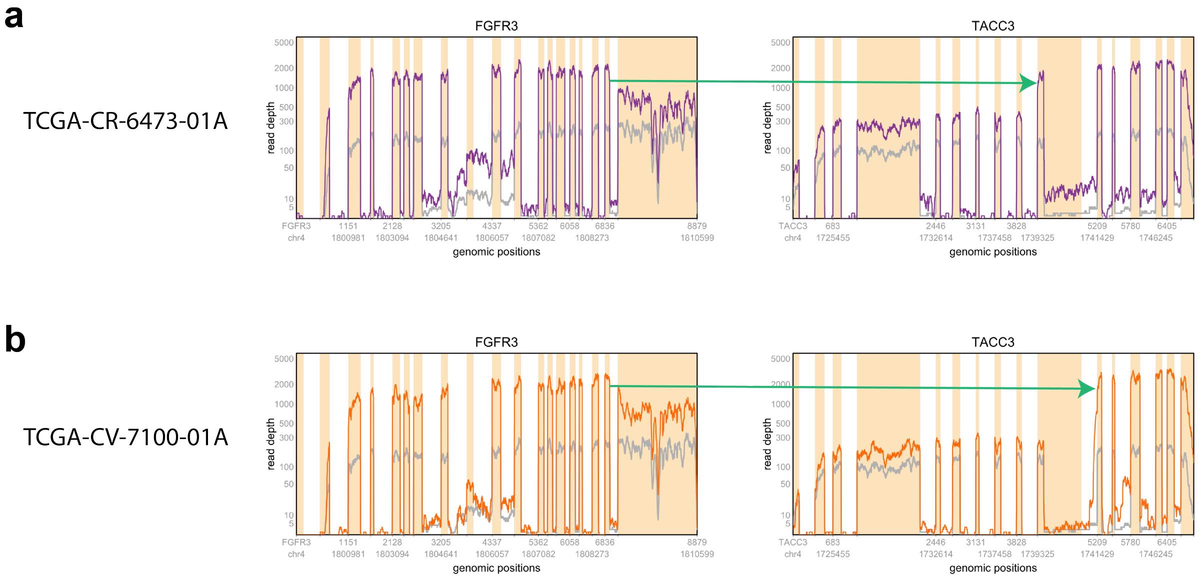

Supplementary Figure 9: *FGFR3-TACC3* fusion events. SCISSOR identified two samples, TCGA-CR-6473-01A (top) and TCGA-CV-7100-01A (bottom), with evidence of *FGFR3-TACC3* fusions, which previously were reported in HNSC. The per-base expressions for each gene locus are plotted (left: *FGFR3*, right: *TACC3*). As shown in the coverage shapes, the fusion events exclude the last exon of *FGFR3* and approximately the half of *TACC3* although splice junctions are different for the two samples. The splice junctions are between Chr4:1,808,661 in *FGFR3* and Chr4:1,739,325 in *TACC3* for the top sample and Chr4:1,741,429 in *TACC3* for the bottom sample. The grey curve indicates the mean coverage across the cohorts (n=452).

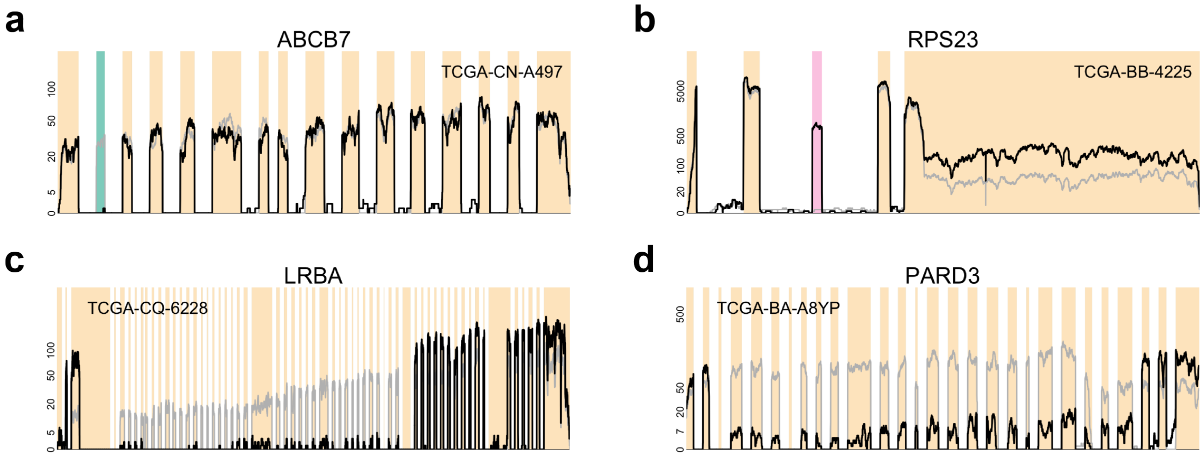

Supplementary Figure 10: Some examples of novel genes from the genome-wide study of SCISSOR. Examples of the shape changes at some of the novel genes in Fig. 3b are presented. (a) As a novel event similar to *MET* exon 14 skipping, a gain of function event previously reported in HNSC, we did observe recurrent exon 2 skipping in 9 samples (2%) in the gene *ABCB7*, a gene known for its aberrant splicing associated with variants of myelodysplastic syndrome. On closer inspection, we noted that each of the samples demonstrating the skipped exon also had a germline intronic variant (NM_004299.6:c.249+1G>A). (b) SCISSOR identified a novel exon of *RPS23* in intron 2 (NM_001025). This 96bp-long exon (pick background) was expressed in three tumor samples but not in normal samples (n=44). No recurrent variants were found. (c, d) For *LRBA* and *PARD3*, a variety of structural variants were identified including deletions of consecutive exons in the middle of the genes.

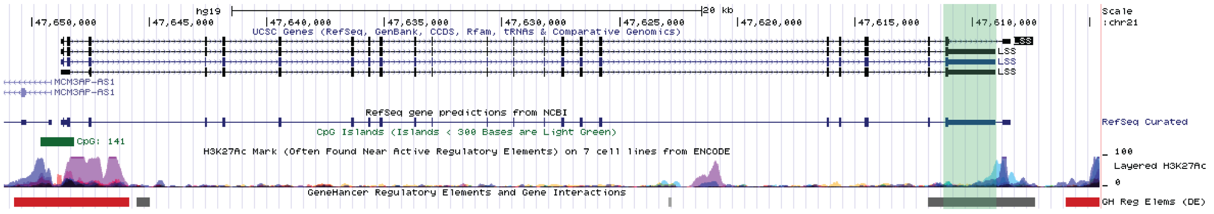

Supplementary Figure 11: *LSS* 3' UTR deletion. Transcripts of *LSS* provided by UCSC genome browser are shown with regulatory elements information. The last exon in the 3' UTR involved in large recurrent deletions in multiple samples (Fig. 3e) is highlighted by a green rectangle. In the bottom, GeneHancer track shows an enhancer with the high confidence score of 356 (GeneHancer id: GH21J046187).

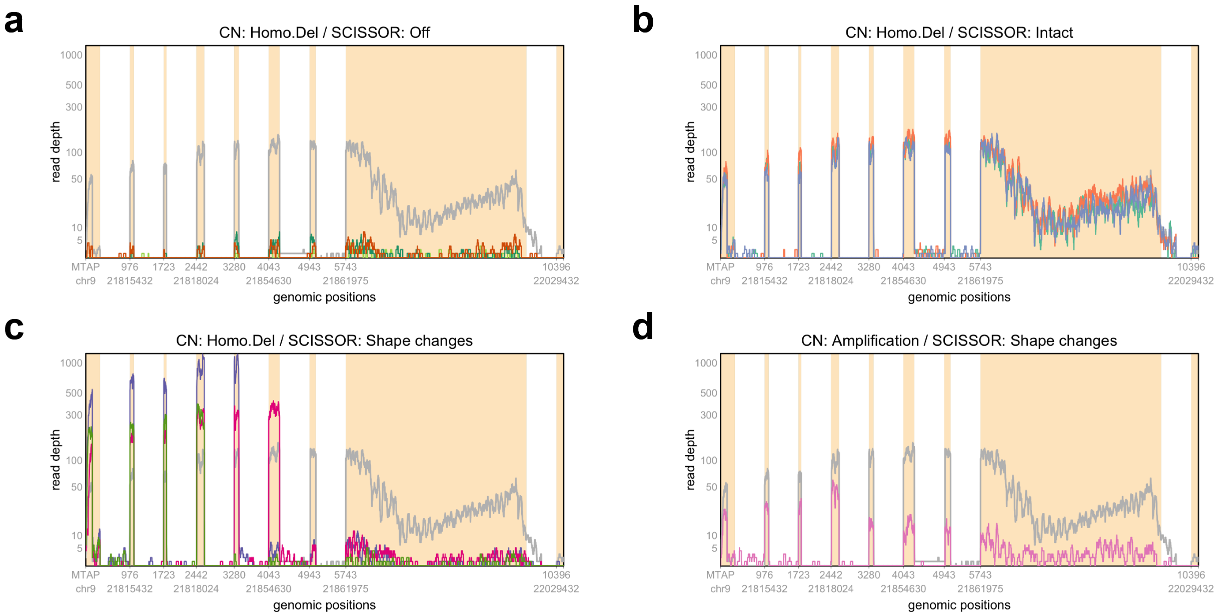

Supplementary Figure 12: Integration of DNA copy number and SCISSOR shape changes for *MTAP*. In *MTAP*, DNA copy number analysis reported 57 samples (12.6%) as homozygous deletions and two samples (0.4%) as amplification (cBioPortal). However, a closer investigation with our shape-based analysis suggested the possibility that many of the samples might have been mis-classified. SCISSOR identified structural alterations in 12 samples (2.7%), and its on/off analysis using the LSS scores further identified 4 off-samples (0.9%) in the samples without shape changes. Panel (a) shows these off-samples. As expected as the result of homozygous deletions, these samples expressed very low read coverage. The gray curve indicates the mean of the base level expression coverage across all samples (n=452). Using the SCISSOR filtering procedure, there were no cases in which SCISSOR classified a gene as “on” while the copy number classified homozygous deletion. For the other samples with copy number abnormalities, however, we observed that they presented either intact (n=42) or structural variants (n=13) in contrast to their designation as homozygous deletion. Panel (b) shows three representative examples of intact samples overlapping the gray curve presenting median overall expression with normal coverage pattern most consistent with intact gene structure. While it is possible that measured gene expression in the context of a call of homozygous deletion could be derived from contaminating normal stroma, we suspect the more likely explanation is misclassification at the boundary of the deletion call. Further, Panel (c) and (d) display examples of the structural variants that were also incorrectly called as homozygous deletions (c), supporting the interpretation that misidentification of the boundary of the deletion might explain at least some cases seen in panel (b). A final example of a sample labeled as amplification is shown to further support the benefit of RNA in correct interpretation of the driver status of a DNA copy number event. The locus may be amplified, but there is no support that the gene is the target of that event. Taken together, these data provide the strongest evidence that MTAP is targeted in a manner consistent with a driver gene mutations with 2.7% structural alterations as well as additional homozygous deletions. The call of homozygous deletion alone, however, includes many false positives and misclassified structural variants which are further characterized by SCISSOR.

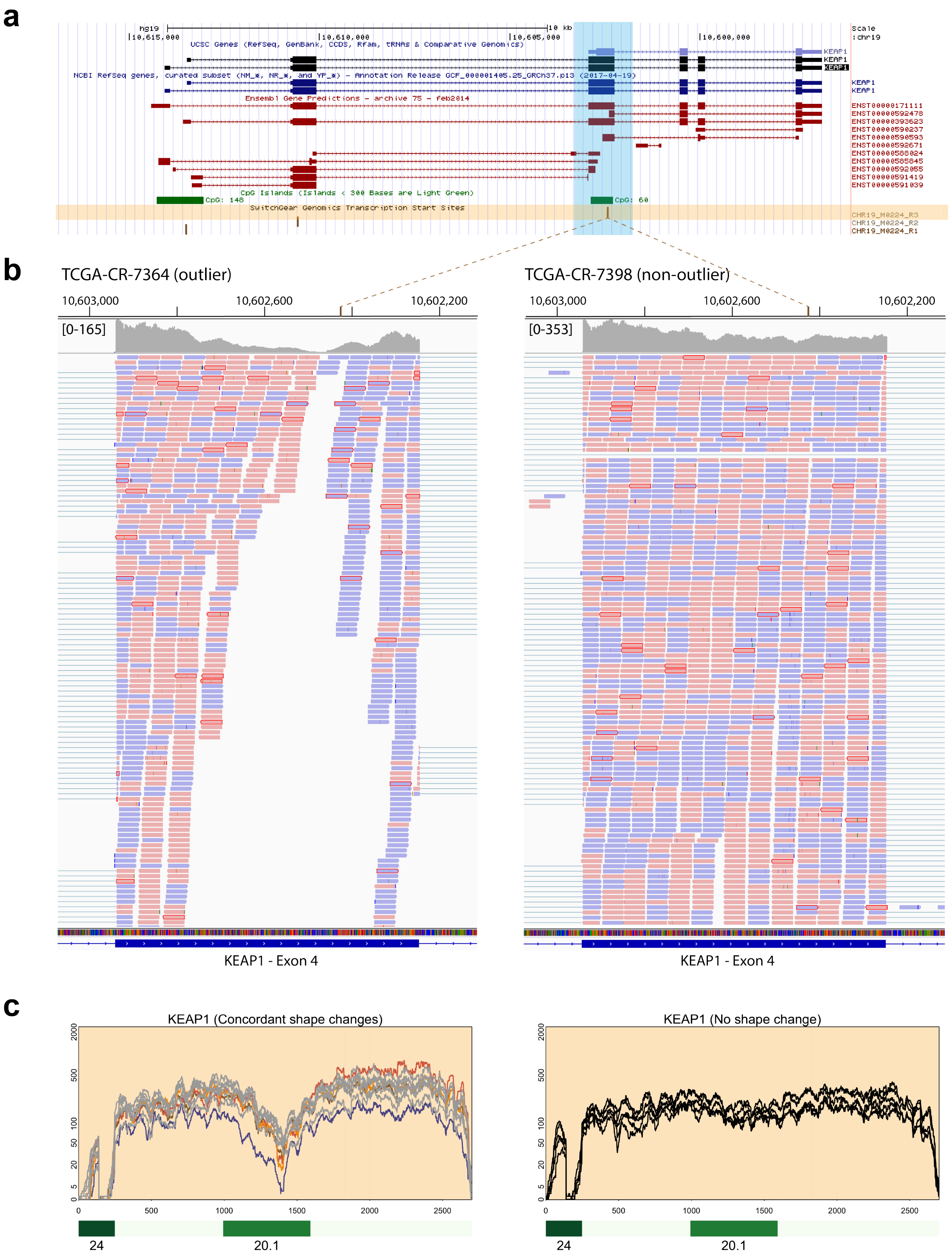

Supplementary Figure 13: Alternative transcription start site (ATS) in *KEAP1*. (Caption next page.)

Supplementary Figure 13: Alternative transcription start site (ATS) in *KEAP1*. (a) Transcripts, CpG islands, and SwitchGear Genomic TSS (transcription start site) for *KEAP1* locus provided in UCSC genome browser are shown. The exon at which concordant shape changes were observed in multiple samples is highlighted by sky blue. Several transcripts that start or end in the middle of this exon have been reported. As indicated by an apricot rectangle at the bottom of the figure, an ATS was reported within this exon by SwitchGear Genomics at chr19:10,602,425 with a confidence score of 15.0. (b) Short reads colored by strand (pink or blue) are shown using IGV for a concordant outlier (left, TCGA-CR-7364) and a non-outlier (right, TCGA-CR-7398). The genomic position of the reported ATS is indicated by the brown tick mark with dashed lines connected to (a). In the middle of the left sample, the empty area exhibits scarce reads consistent with read deletions observed in concordant outliers shown in Fig 4b. As indicated by reads colors, interestingly, densities of pink and blue reads are different on each side of this empty area whereas they are almost evenly distributed on the right sample. Such uneven proportion of different read strand together with a relatively small number of overall reads is often observed at the end or start of a transcript because one of paired reads is unavailable at the very ends of a transcript. Therefore, this suggests a strong evidence of an alternative transcription initiated at this region. On the other hand, the equally distributed reads in the right panel strongly indicate that the corresponding region is a gene body for the particular sample. (c) Coverage shapes of *KEAP1* for the identified concordant outliers and non-outliers are compared. Left, the four concordant outliers that were co-altered in a large number of genes and strongly correlated by the Fisher's exact test are shown in different colors. Some additional weak outliers by a targeted review are shown in grey curves. Right, some samples with no shape changes are shown in black curves. Taken together, this is consistent with abnormal transcription of this gene at which two non-contiguous transcripts are expressed rather than one continuous transcript.

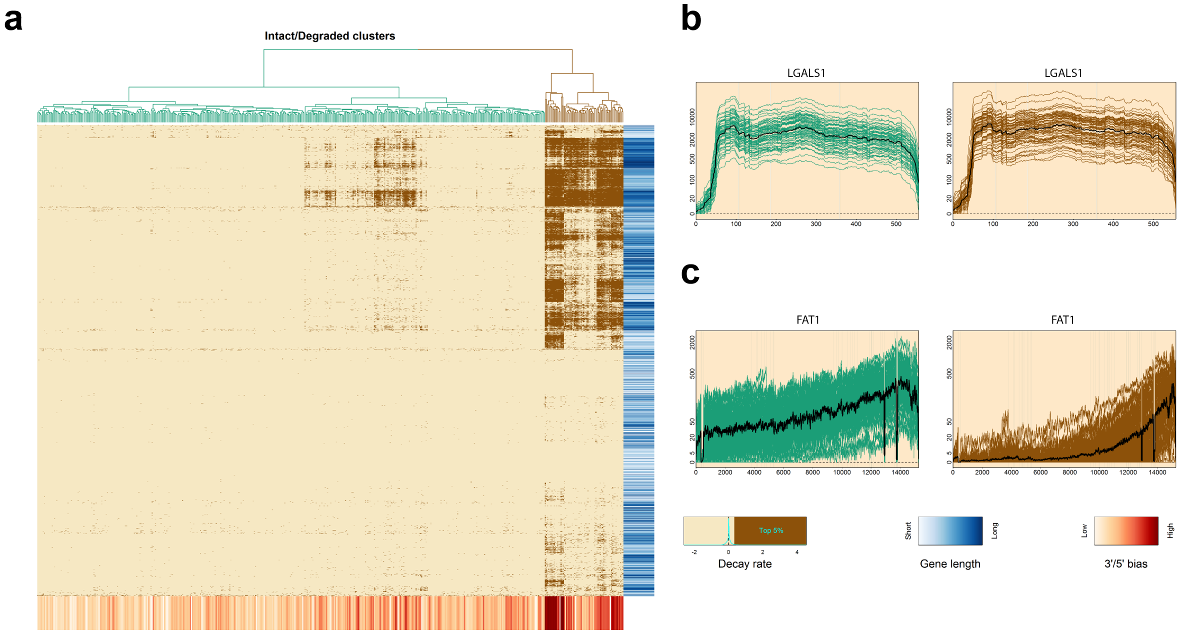

Supplementary Figure 14: Decay rates and identified degraded cases. (a) Heatmap of decay rates for all genes and all tumor cases, in which the brown color indicates the top 5% decay rates whereas the beige color indicates the lower values. The vertical color bar on the right of the heatmap encodes the gene lengths and a darker blue denotes a longer gene. The horizontal color bar on the bottom of the heatmap encodes the decay ranks from the 3'/5' bias method and a darker red denotes higher decay rank [2]. Based on two groups from the hierarchical clustering on the top of the heatmap, the 70 identified degraded cases are in brown and the rest of intact cases are in green. (b) Log10 RNA-seq for a short gene, *LGALS1*, which is 556 bp long. (c) Log10 RNA-seq for a long gene, *FAT1*, which is 15,262 bp long. In (b) and (c), a set of intact cases are shown on the left plots and all identified degraded cases are shown on the right plots with the mean indicated by the thick black curve.

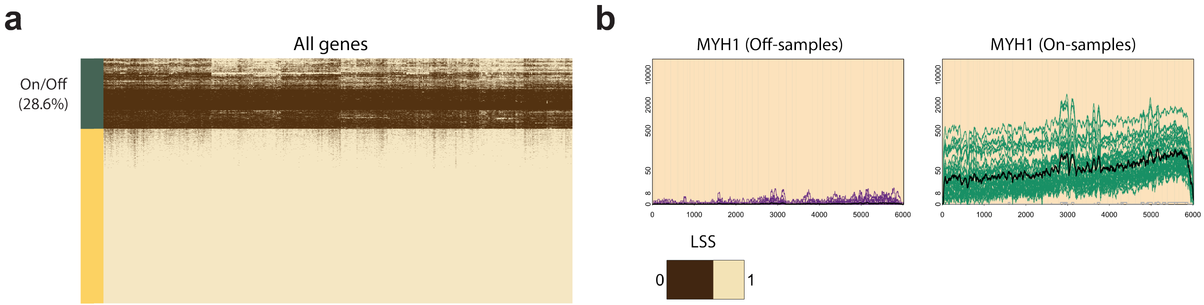

Supplementary Figure 15: Gene filtering. (a) Heatmap of the binary matrices from the level of shape-similarity (LSS) are shown. If the LSS is greater than 0.6, the value is 1. Otherwise, the value is 0. The rows are all genes and the columns are all cases. The 5802 genes (28.6%) were identified as the genes violating the assumption of SCISSOR. (b) An example (*MYH1*) of the identified genes to be filtered. The two plots show the difference between two groups (Expressed/Unexpressed) in the sense that the green samples are well-expressed at *MYH1* whereas the brown samples are under-expressed.

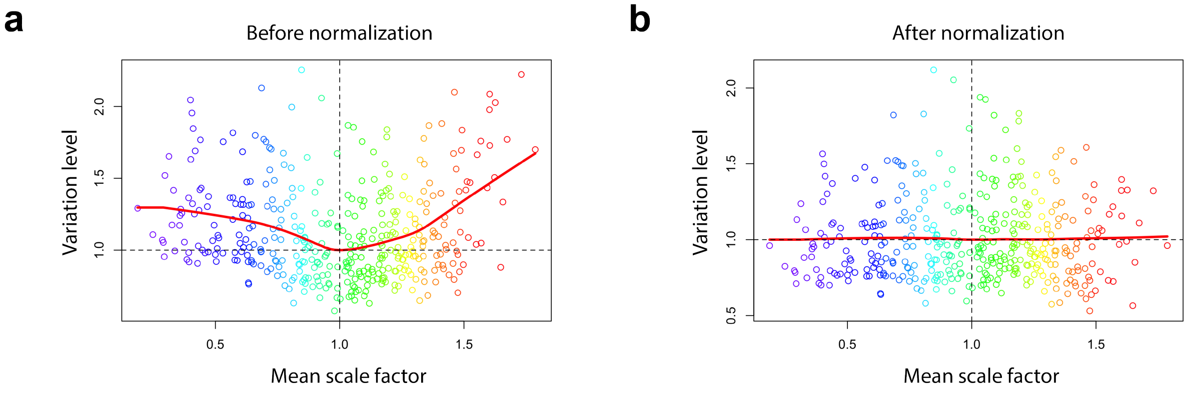

Supplementary Figure 16: Comparison of sample variation before and after normalization. Overall variation levels with respect to overall expression levels for gene *TP53* before and after applying normalization. Each point represents each sample and the rainbow colors indicate different levels of expression measured by the mean scale factor. The red curves are the estimates using a smoothing technique LOWESS with a smoothing parameter of 0.7. (a) The data before normalization clearly show systematic variabilities with respect to overall expression levels. (b) After normalization, the overall variations become independent of overall expression levels showing no particular patterns.

Supplementary Note 1: Normalization

Numerous normalization approaches for RNA-seq have been proposed [3, 4, 5, 6, 7, 8, 9, 10, 11]. However, these approaches were designed to standardize gene expression data at the genome-wide level often by scaling the total number of reads across samples. A recent study has considered normalization at the base-level to identify clusters of differential isoform usage [12]. They filtered out low-expression samples and performed count normalizing followed by log-transformation. However, biologically important shape changes are still observed in low-expression samples, and simply removing low-expressed samples may lose important genetic events. Also, scaling each remaining sample by its total expression can distort its genuine shape architecture by yielding unwanted fluctuation especially in lowly-expressed regions. Such artificial variation may confound other important variation. Here, we propose an effective way to standardize base-level read coverage data between samples at a given gene, while preserving important shape deviation.

Our group and others have previously shown that when the read counts are low or highly variable, estimates of gene expression have high variance compared to genes with higher expression. To avoid spurious detection of differences, investigators must either remove such genes by filtering or adopt variance stabilization approaches such as pseudocounts[3-5]. The use of pseudocounts are preferred since solutions have been proposed to model stabilized variance, such as assessment of symmetry around the median. Additionally, variance stabilization obviates the need to discard genes through filtering[2]. Whereas most studies parameterize pseudocounts at the experimental level by selection of a single offset value, our purpose of characterizing gene by gene shape changes requires variance stabilization at the gene level. For the purposes of variance stabilization we optimize the A-D statistic as a function of pseudocount offset.

Algorithm for a proper log-transformation. Supplementary Figure 17 (a) shows the raw coverage overlays of every sample at the gene *CASP1* using the pileup format as an example clearly presenting unstable dynamics such as strong skewness and different variabil-

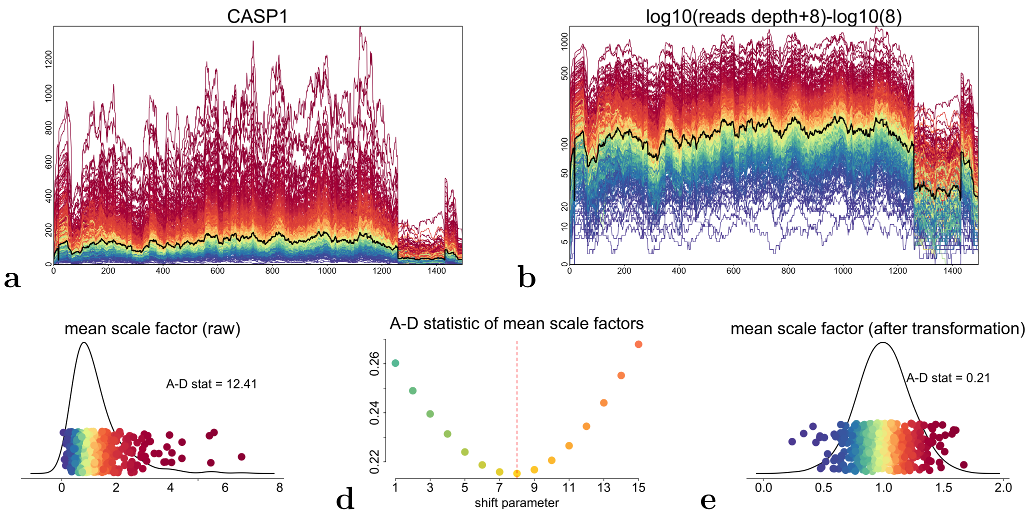

Supplementary Figure 17: Automatic data transformation at gene *CASP1* using pileup format. (a) The raw coverage data for all patients (n=452) are shown using rainbow colors, which correspond to the mean scale factors in (c). The red color indicates highly expressed samples and the purple color for lowly expressed samples. The black curve represents the trimmed mean across samples at each base. The data show severe skewness and heterogeneous variation across samples and bases. (b) The log transformed data with the automatically selected shift parameter of 8 are shown with the same rainbow color scale as (a). The black curve represents the trimmed mean across samples at each base. The transformed data are roughly symmetric about the mean curve and show stable variation across samples and bases. (c) The mean scale factors from raw coverage data are shown with the kernel density estimate using the black curve. The distribution is skewed and the A-D statistic of 12.41 indicates strong skewness. (d) The A-D statistics with respect to a shift parameter from 1 to 15 are shown. The value 8 produces the smallest A-D statistic. (e) The mean scale factors with the transformed data using the parameter value 8 are shown with the kernel density estimate. The distribution seems roughly symmetric about the mean 1. The A-D statistic also shows significantly reduced skewness compared to (c).

ities across bases as well as across samples. Log-transformation stabilizes such heterogeneity and reduces the extreme skewness of raw counts, but different log shift parameters result in different levels of stabilization. Thus, it is expected that a careful selection of the parameter helps to obtain a less skewed and roughly symmetric data distribution. Also, different genes present different dynamics in coverage, so it is desirable to apply a more suitable parameter for each gene. In this section, we propose an automatic procedure to determine a shift parameter for each gene.

The proposed algorithm is summarized as follows:

1. For a range of parameters, say $1, \cdots, 10$, consider candidates (e.g. integers) of the shift parameter that will be considered in the grid search and compute the Anderson-Darling (A-D) statistic for each candidate based on the following steps.
   (a) Take the log-transformation of the data with the given parameter.
   (b) Obtain MSF_j ($j = 1, \cdots, n$).
   (c) Compute the A-D statistic of $MSF_1, \cdots, MSF_n$.
2. Select the parameter which gives the smallest A-D statistic.

Supplementary Figure 17 (c) shows the mean scale factors of the raw counts data in (a) with the kernel density curve. The skewed curve as well as the corresponding A-D statistic of 12.41 support that the distribution is substantially skewed. Supplementary Figure 17 (d) shows the A-D statistics with respect to the parameter candidates $(1, \cdots, 15)$. Although any positive values can be used, we considered only positive integer values for the faster computation and easier interpretation. Also, for the genome-wide analysis, we limited the range of parameters up to 10. This is because it has been observed that the shift parameter values beyond 10 often produce too little variation for low counts compared to high counts, which may result in shape changes at low expression levels being less detectable. Supplementary Figure 17 (d) indicates 8 as the chosen shift parameter, and the log transformed coverage data with the chosen parameter are shown in Supplementary Figure 17 (b). It can be seen that the variation is now nicely stabilized within samples as well as between samples. The corresponding MSFs and the A-D statistic shown in Supplementary Figure 17 (e) also attains much less skewness of the distribution than before the log transformation.

Mean scale correction. As discussed, the proposed model is designed to account for the systematic variation from technical biases via sample-specific scaling factors a_j and a smooth curve $g(a_j)$. The a_j's describe the different levels of overall expression and $g(a_j)$ accounts for the remaining unwanted variation subject to the expression levels. The key aim of the mean scale correction is to estimate these unknown parameters and get rid of them in order to get the normalized data x_{ij}. Based on the observed R_{ij}, the other unknown elements μ_i, a_j and $g(.)$ can be estimated as described below.

First, we estimate μ_i by a trimmed mean at each base-position. This simple method effectively down-weights possible outliers and helps to robustly estimate the true mean expression levels. Next, as the majority of samples share the overall shape patterns with different expression levels, the a_j can be estimated by using the linear model. Then, we equate the overall expression levels of samples to zero by subtracting out the sample-specific overall expression as follows:

$$r_j = \log R_j - \hat{a}_j \log \hat{\mu}.$$

where $\hat{a}_j$ and $\hat{\mu}$ are the estimates of a_j and μ.

Now, we propose a procedure to estimate the unknown function $g(a)$ that describes overall variation subject to the expression levels a_j. Suppose that a_j and μ are known and they are normalized out. Then, our model becomes

$$\log R_j - a_j \log \mu = g(a_j) X_j.$$

Letting $v_j = \log R_j - a_j \log \mu$, we have the following relationship:

$$\sum_i v_{ij}^2 = g^2(a_j) \sum_i x_{ij}^2.$$

By taking the expectation to both sides, we have

$$\begin{aligned} E[\sum_i v_{ij}^2] &= g^2(a_j) E[\sum_i x_{ij}^2] \\ &= g^2(a_j)\tau \end{aligned}$$

where $\tau = E[\sum_i x_{ij}^2]$. This implies that if $g(.)$ is independent of the scale factor, i.e. $g(.)$ is a constant function, then $\sum_i v_{ij}^2$ should be centered at τ with some variation with no systematic pattern. Conversely, if $\sum_i v_{ij}^2$ shows a certain systematic pattern with respect to a_j, then it can be an evidence of variation subject to overall expression. The characterization of this pattern thus can be used for the estimation of $g(.)$. However, there is an unknown factor τ, indicating that $g(.)$ is unidentifiable solely based on $\sum_i v_{ij}^2$. Let $f(a) = g^2(a)\tau$. By giving a constraint $g(1) = 1$ as stated before, we have $f(1) = g^2(1)\tau = \tau$, allowing $g(a)$ to be identifiable by $\sqrt{f(a)/f(1)}$. The remaining part is then the estimation of $f(a)$.

Using the estimates from above, we can replace the v_j by $r_j = \log R_j - \hat{a}_j \log \hat{\mu}$ and thus $r_j^T r_j = \sum_i r_{ij}^2$ can be used to estimate $f(a)$. Simply taking the sum of squared values from data may not be a good estimate because it can easily break down due to some extreme values. This limitation can be overcome by employing other robust approaches. Here, we use the Tukey's bisquare method to downweight extreme values, allowing a more stable estimation, but other robust methods can be also used. Based on Tukey's bisquare function ρ, i.e.

$$\rho(r) = \begin{cases} \frac{k^2}{6}\left\{1 - [1 - (\frac{r}{k})^2]^3\right\} & \text{for } |r| \leq k \\ \frac{k^2}{6} & \text{for } |r| > k, \end{cases}$$

we estimate the $E[\sum_i v_{ij}^2]$ by

$$\sum_i \rho(r_{ij})$$

with the tuning parameter $k = 4.685\sigma$ where σ is the sample standard deviation of r_{ij}.

Supplementary Figure 16a shows the properly scaled $\sum_i \rho(r_{ij})$ with respect to $\hat{a}_j$ at gene *TP53* such that the center of the points is $(1, 1)$. The points exhibit a systematic pattern, which supports the existence of non-negligible impact of overall expression onto the variation r_{ij}. The function f can be estimated by pooling information across points and fitting a smooth curve to capture the global trend. Then, the function g is estimated by the fitted curve $\hat{f}$ scaled by $\hat{f}(1)$ so that $\hat{g}(1) = 1$. The estimated function $\hat{g}$ for the gene *TP53* is shown by the red curve. We divide r_{ij} by $\hat{g}(\hat{a}_j)$, i.e. $\frac{r_{ij}}{\hat{g}(\hat{a}_j)}$, to remove the overall expression-dependence. We repeat this procedure until we get the data object whose pattern is not systematic any longer. The adjusted variation levels after the normalization are shown in Supplementary Figure 16b.

Supplementary Note 2: *TP53* splice site mutations in HNSC

A splice site mutation is defined as a base substitution that occurs in either the donor or acceptor site relevant to the processing of pre-mRNA to mature mRNA. As a result of

disrupted splice sites, the abnormal mRNA may remain introns, skip exons, or activate alternative splice sites. In the HNSCC example, there were 39 splice site mutant samples in *TP53*, of which SCISSOR reported 23 samples as significant shape changes (Figure 2). We then asked if the remaining 16 samples have any evidence of altered splicing by cause of splice site mutations.

To evaluate the effect of a splice site mutation on abnormal splicing in each sample, we performed an abnormal read-ratio analysis. We first extracted reads (A) mapped to the splice site containing the mutant base (or adjacent splice site) and computed the ratio of abnormal reads (B) to the collected reads, i.e. B/A. To determine the types of abnormal splicing, the abnormal reads were further classified into two groups: reads abnormally retained in an intron/intronic part (B1) or the ones skipping an exon/exonic part (B2). If the ratios B1/A or B2/A are unusually large, then we might conclude that the mutation under consideration is associated with intron-retention or exon-skipping, respectively. For an abnormally retained read, it is required to cover ≥ 5 bp on both exon and intron.

We applied the abnormal read-ratio analysis to the 16 samples, and the results were summarized in the table. We observed that 6 of them had no evidence of abnormal splicing with zero abnormally spliced reads. In general these samples also documented low expression overall. We interpret this to mean that either the spice site resulted in a loss of the spliced allele, through a process such as nonsense mediated decay, or that perhaps a second event had further impacted the expression of the gene. Finally, abnormally spiced reads might be difficult to map, although if this were the case, the SCISSOR method would be more likely to identify the abnormal shape generated by loss of mapped reads. Interestingly, the phenomenon of spice site mutation without evidence of variant splicing was not commonly observed in other tumor suppressor genes in this tumor type such as *CDKN2A* or *FAT1*. In an additional 6 samples with spice site mutation, there was a similar but less extreme example of the same phenomenon in which spice site mutations were associated with either detectable but very low allele fraction of the abnormally spiced reads ($< 10\%$) relative to total reads or overall extremely low gene expression (1 percentile based on a normalized RSEM). The remaining 4 samples showed fairly high ratios of abnormal reads and we further interrogated these samples to understand the inconsistent results with SCISSOR (Supplementary Figure

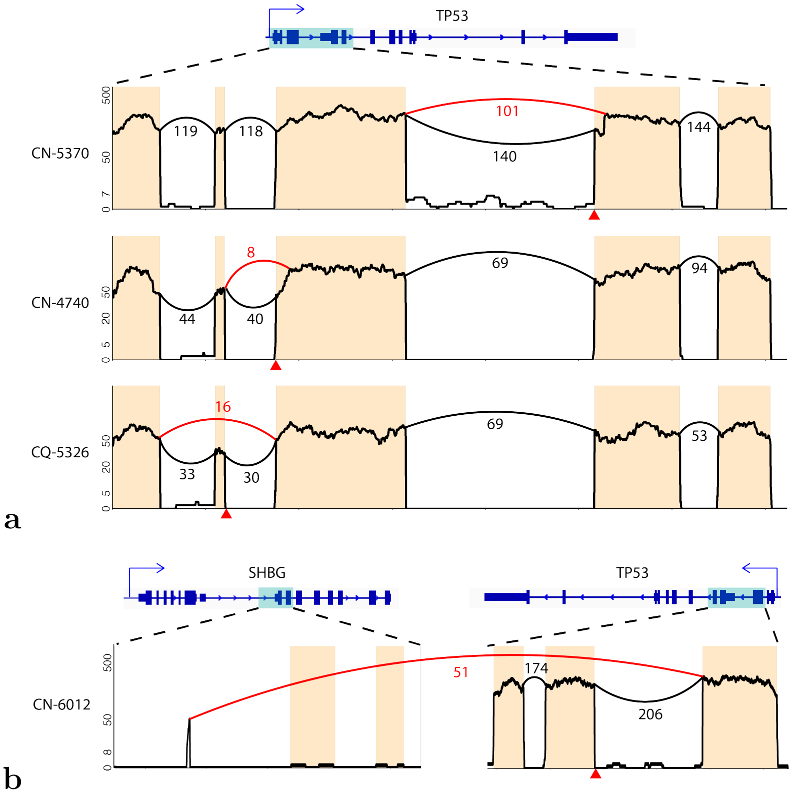

Supplementary Figure 18: *TP53* splice site mutations disrupting splicing but missing from SCISSOR. In each sashimi plot, a red triangle below the x-axis indicates the site at which the splice site mutation occurred and a red junction curve indicates abnormal splicing. (a) The three samples CN-5370, CN-4740, and CQ-5326 present their coverage expression profile for the first 5 exons of *TP53* (roughly, chr17:7,578,000-7,580,000) indicated by the highlighted region of the transcript above them. (d) The last sample CN-6012 shows the parts of *TP53* and *SHBG* loci where abnormal junctions are mapped.

18).

For CN-5370, 42 of reads were abnormally spliced. These abnormal reads all skipped the 5' aspect (21 bp) of the 4th exon, which gives an evidence of an alternative 3' splice site activation. Similarly, CN-4740 also had an evidence of an alternative 3' splice site activation (19 bp) although the ratios of abnormal reads were relatively low ($\sim$ 17%) and the gene expression were relatively low. We suspect that these 2 cases represent false negative for SCISSOR. The small size of the skipped region, relative to the window size of 50 used in this paper for the step 2 procedure may be responsible for the failure. However, it is expected that this type of missing events could be identified by using a different window size or a different approach of localization. Alternative results by using a different window size might

be considered as a limitation of SCISSOR and further discussed in the section of Discussion.

Another sample CQ-5326 had $\sim 33\%$ abnormal reads which skipped the short 2nd exon (22 bp long). This exon was shorter than the read length (50 bp), which possibly divided a read into three split junctions and thus made it harder to align these split junctions onto the genome. We observed that this resulted in significantly low coverage on the 2nd exon in the majority of samples. As the 2nd exon is relatively low overall compared to the other exons, the reduced expression due to abnormal splicing is less distinguishable based on coverage.

The last sample CN-6012 had $\sim 20\%$ abnormal reads that skipped all the exons from the 4th. The two split junctions from each of the abnormal reads were aligned to the end of the 3rd exon of *TP53* and an intronic region starting at chr17:7,533,126 of the gene *SHBG* located ahead two genes from *TP53*. This event was missing from SCISSOR because of the shrinkage effect of logarithmic transformation for large values. That is, the difference between two large values becomes smaller compared to the difference between two small values. This shrinkage effect can make a moderate level of deletion in a highly expressed sample less noticeable as a shape variant. SCISSOR uses logarithmic transformation, with an automatically chosen shift parameter in the normalization step, because it has multiple well-known advantages such as variance stabilization of raw counts, a simple interpretation, and a linear correlation with sequencing depth. To help this type of event more distinguishable, however, a different transformation might be employed but it might lose some good properties of logarithmic transformation. Therefore, it is an interesting future direction to address some unfavorable properties of logarithmic transformation in RNA-seq shape variant detection.

We conclude that while SCISSOR performed well overall in those cases where a spice site mutation resulted in altered spicing as measured by short read aligned data, there may be some room for improvement in future implementation. We also observed, that in some cases spice site mutations may be mischaracterized. Most importantly, variant annotation software will often assign the most significant annotation to a base substitution from among all possible transcripts. If that high-impact event is incorrectly annotated as spice site rather than missence, then SCISSOR can provide some evidence as to this error.

Supplementary Note 3:

Methods comparison

We evaluated SCISSOR along with several relevant methods for detecting alternative splicing events [3, 13, 14, 15, 16, 17]. The most common data types used for this task are exon-level expression (expression represented by the number of reads aligning to each exon) and junction reads (counting split reads aligning to separate exons). Although SCISSOR is an unsupervised outlier detection method designed to interrogate expressed gene variants across the entire gene, biologic interpretation of gene outliers is often in terms of alternative splicing or exon expression at a single locus or region. Detection of alternative splicing and exon expression is the outcome of many supervised RNA-seq algorithms, and as such, we considered a comparison of the results of SCISSOR versus more common approaches to detection of exon skipping and alternative splicing. As a direct comparison was not obvious, given differences between unsupervised methods and supervised methods, algorithmic modifications and normalization required to articulate an objective comparison. However, it was possible to adapt the statistical framework underlying the two most common approaches to alternative spicing and gene expression using junction reads-based analysis (JRBA) and exon level expression-based analysis (ELBA). JRBA and ELBA are explicitly involved as the framework for most if not all relevant comparator methods including the percent-spliced-in [13, 14, 15, 16, 17] and DEXseq [3]. We adapted some of these methods to obtain exon-level expression and junction reads data amenable to the outlier detection and compared the results from each data type, JRBA and ELBA, with SCISSOR.

At a single gene TP53

Exon level expression based analysis (ELBA). For ELBA, we adapted DEXSeq which is a tool to test for differences in exon usage between two groups of samples. It makes use of exon-level expression at a single gene by counting the reads mapping to the flattened exons (bins), modeling them using a negative binomial distribution, and inferring changes based on the generalized linear model. Preparing annotation and counting reads were processed using

Python (version 2.7.4) as suggested in the DEXSeq package. DEXSeq stands out from other similar approaches in that its normalization procedure helps to effectively remove possible confounding signature particularly resulted from different library sizes. For the purpose of detecting abnormal exon usage, it is critical to get a data object that is free from other confounding factors.

Therefore, we applied the normalization procedure of DEXSeq to obtain the exon-level expression data for *TP53* locus normalized by using 183 genes known as tumor suppressor genes. This resulted in 36 exon bins (for *TP53*) each of which contains normalized read counts for the cohort. Five out of the 36 exon bins were excluded owing to their scarce reads in every sample. Taking the logarithmic transformation of normalized counts from 31 exon bins for a data object, we performed the projection outlyingness approach implemented in SCISSOR to detect sample outliers involved in abnormal exon usage (Methods).

The resulting outlyingness scores are shown in Supplementary Figure 19 (b). The 19 outliers were identified with the significance level 10^{-4} as used in SCISSOR, and many of them were associated with significant exon skipping events such as whole exon skipping or multiple partial exon-skipping. Because the data are tuned in for the exons from pre-existing transcript annotations, however, this approach is limited to detecting abnormal changes in these exons and cannot discover novel alternative splicing events or changes in intronic regions. Besides this inherent limitation, the most critical issue might be that it was unsuccessful for detecting moderate level of exon-skipping events as well as deletions within an exon, which skipped only a limited fraction of exon reads. This is mainly because pooled counts represented by each exon coverage using a single number cannot describe full dynamics of read coverage compared to base-level representation.

Junction reads based analysis (JRBA). For junction reads data, the percent-spliced-in, often denoted by ψ, has been commonly used for determining whether an exon is included or excluded [15, 18]. Although each method targets different types of alternative splicing, however, many of them do not consider the inclusion of introns which is a common form of abnormal splicing. This is mainly because intron retention cannot be revealed by split reads or exonic reads used in most of junction-based analysis, and rather reversely, it entails

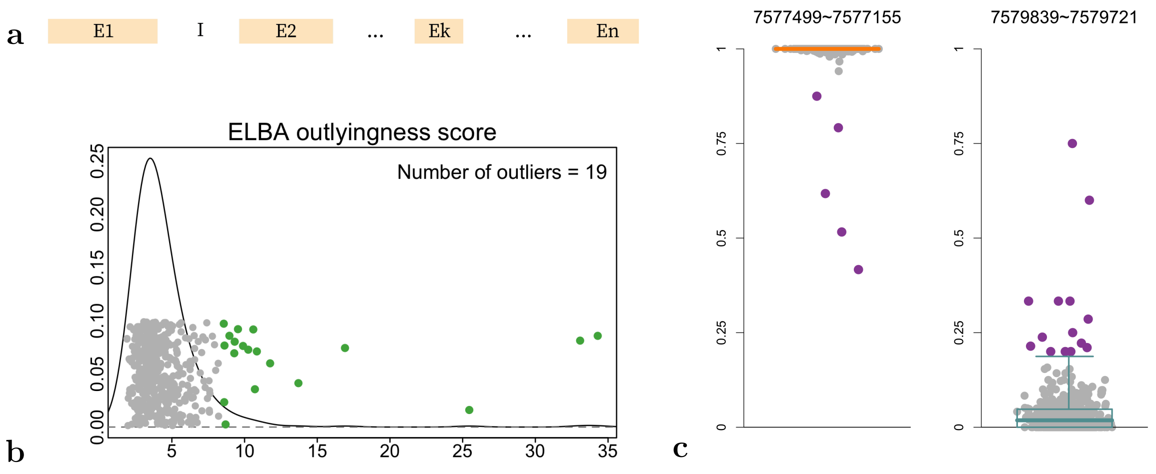

Supplementary Figure 19: ELBA and JRBA. (a) A hypothetical gene model. Colored bars are exons and white bar indicates intron. (b) The projection outlyingness scores from ELBA with a kernel density estimate. The 19 outliers identified based on the significance level $\alpha = 10^{-4}$ are indicated by the green. (c) Distributions from two selected splice junctions are shown with box and whisker plots with the median, interquartile range (IQR), and $c\times$IQR distances from the upper and lower ends of the box marked, where the c was determined by a pre-determined significance level= 10^{-4}. The outliers based on the same significance level are indicated by the purple. Left, the values of ξ_{AE}'s for the junction between (7577499, 7577155) are shown. The outliers from this junction are potentially associated with exon skipping event because every sample but the five outliers has almost 100% of splice reads split into this junction. Right, the values of ξ_{IR}'s for the junction between (7579839, 7479721) are shown. The outliers from this junction are likely to be associated with the intron retention event because of the high percentage of retained reads.

retained reads spanning an intronic region (Figure 1). For this reason, we propose a new method which is adapted from the ratio-based splicing (RS) analysis [13] to cover intron retention as well as alternate exon usage for outlier detection in an unsupervised setting.

To look for splice junctions, we first extracted split reads that partly or fully aligned to *TP53* locus from bam files generated by Samtools. From this set of split reads, we annotated all possible splice junctions by taking the positions which the reads were split into. Total 599 splice junctions were found, which was a much larger number than the 10 junctions annotated in known transcripts. To address the disparity between a large number of junctions relative to the known exon models, we performed the following procedure. Denote a read spanning the junction between A and B by $R_{A\sim B}$. If both of A and B are exons, $R_{A\sim B}$

indicates a split read aligning to exon-exon junction. On the other hand, if one of A and B is an intron (adjacent intron of the other), $R_{A\sim B}$ indicates a retained read of full length spanning the exon-intron junction. For accuracy, we only consider a retained read covering $\geq$ 5bp on both the exon and intron side. We propose the following two different measures each of which targets different abnormal splicing event (Supplementary Figure 19 (a)):

(a) For intron retention: To determine if an intron I is abnormally retained, we use the fraction of retained reads, i.e. $\xi_{IR} = \frac{\#R_{E1\sim I}}{\#R_{E1\sim I}+\#R_{E1\sim E2}}$ for $\#R_{E1\sim I} + \#R_{E1\sim E2} \geq 2$ and $\xi_{IR} = 0$ otherwise.

(b) For alternate exon: To determine if an exon E^* is abnormally excluded or included, we use the fraction of split reads aligning to $E1 \sim E^*$ respect to all split reads from E1, i.e. $\xi_{AE} = \frac{\#R_{E1\sim E^*}}{\#R_{E1\sim E^*}+\sum_{k=1}^{n}\#R_{E1\sim Ek}}$ for $\#R_{E1\sim E^*} + \sum_{k=1}^{n}\#R_{E1\sim Ek} \geq 2$ and $\xi_{AE} = medianofother\xi'_{AE}s$ otherwise.

We applied (a) and (b) to every annotated junction across the cohort ($n = 452$), resulting in two sets of values, n ξ_{IR}'s and n ξ_{AE}'s, for each splice junction. From each of the distributions, an unusually high or low value compared to the majority of values was determined as a splicing outlier. As the values are proportions between 0 and 1 and most of the values are concentrated in either end, the resulting distributions are often extremely skewed, which makes it harder to distinguish outlying samples from normal samples. To address this issue, we propose a robust outlier detection procedure which treats separately either side of the median value (Q_2) which enables to reflect different spread of the values on either side. The basic idea is similar to the well-known IQR-based outlier detection rule, but the IQR, i.e. $Q_3 - Q_1$, is replaced by $IQR_L = 2 * (Q_2 - Q_1)$ for the values less than the Q_2 and $IQR_R = 2*(Q_3 - Q_2)$ for the other side. Note that IQR_L and IQR_R are the same as the IQR for a symmetric distribution. We then declared the values less than $Q_1 - c * IQR_L$ or greater than $Q_3 + c * IQR_R$ as outliers with the c determined by a pre -determined significance level (10^{-4} for here). To ensure a sufficient number of abnormal reads, at least 10% of abnormal reads were required to be called as an outlier (Supplementary Figure 19 (c)).

As the first round of analysis, we looked at the 197 junctions (out of total 599) where at least two reads were aligned in total across samples as a basic filtering procedure. This

produced 247 outliers out of 452 samples ($\sim 55\%$), mostly detected from new splice junctions not included in known gene models. Although the ability of JRBA to include unknown splice junctions is helpful for discovery of novel splice junctions, this result suggests that it can also produce a large number of junctions which is a challenge to the biologist for interpretation and prioritization and which might include a large number of false discoveries.

Therefore, some caution is needed in choosing the junctions that will be included. In this analysis, we filtered the junctions that fell outside the *TP53* locus (chr17:7571720-7579940) based on the union of the existing transcripts, which resulted in 42 splice junctions to be considered. From the 42 junctions, we identified 77 splicing outliers many of which were associated with splice site mutations including three missing ones (TCGA-CN-5370, TCGA-CN-4740, and TCGA-CQ-5326) from SCISSOR (Supplementary Figure 18 (a)). These samples were involved in cryptic splice site activation or exon skipping with changes in a very short region of the locus, proving the ability of JRBA to capture novel splice junctions. As shown in the first round of analysis, however, a careful selection of splice junctions should be pre-performed to avoid many false discoveries especially for genome-wide analysis. One of the biggest challenges might be to distinguish the junctions supporting alternate exons from the ones with background noise because the resulting distributions from two cases might be similar.

We found that many of JRBA outliers had low gene expression (Figure 5), which is related to a critical issue of junction-based algorithms including ψ that produce less reliable estimates for lowly-expressed samples or for genes with overall low expression. This issue is a limitation for analysis using proportions as a data object. This approach also failed to identify moderate level of exon skipping and intron retention events. Similarly to the ELBA, a single number representation, which is a proportion in this case, of abnormal splicing makes it harder to capture this more challenging abnormality.

Simulation study

SCISSOR was benchmarked on simulated data to estimate sensitivity and the false discovery rate (FDR) under various settings and to compare SCISSOR to JRBA which was adapted from the well-known measure 'percent-spliced-in' to be suitable for detecting outliers as men-

tioned earlier [13, 14, 15, 16, 17]. As shown in the earlier section with the gene TP53, ELBA generally failed to detect intron retention events and suffered from many false discoveries at low gene expression levels. In this section, therefore, we limited our simulation study to the comparison between SCISSOR and JRBA. We also considered only splicing outliers for a better comparison between the two methods because JRBA is only applicable for splicing outliers. However, we note that SCISSOR can identify a wide range of shape changes beyond the splicing outliers such as indels, alternate transcript initiation/termination, on/off outliers, and previously unknown shape changes as presented in Figure 3 (e, f), Figure 4, and Figure 6.

Simulation settings

For the purpose of creating a simulation study we relied on the tool "Polyester"[19]. Polyester allows the user to generate simulated RNA-seq reads based on an underlying experimentally observed distribution, but which also demonstrate properties desirable for the comparison of competing computational algorithms such as under consideration in the current study. Polyester allows the user to introduce reads at varying allele frequencies that simulate alternative splicing or other structural alterations relevant to our efforts. The experimentally derived source sequence was derived from randomly selected genes – 200 genes for each coverage level – from the hg19 genome. Three different types of RNA-seq shape changes – exon-skipping (ES), intron-retention (IR), and alternative exon (AE) – were simulated with randomly chosen exons and introns for each gene. We considered different VAF, variant allele frequencies involved with shape changes, and different percentages of outliers present in a given gene. Paired-end reads with length of 100 were simulated using Polyester and all simulated reads were mapped to genome using TopHat (version 2.0.14.).

For each scenario, 200 samples were simulated including outliers. We considered 5%, 2.5% and 0.5% for different percentages of outliers each of which indicates that the number of outliers is expected to be 10, 5, and 1. Each scenario is denoted by combining its scenario settings. For example, ES100-10 represents the scenario with an exon-skipping of 100% VAF in 10 outliers whereas IR50-5 indicates an intron-retention of 50% VAF in 5 outliers.

Simulation results

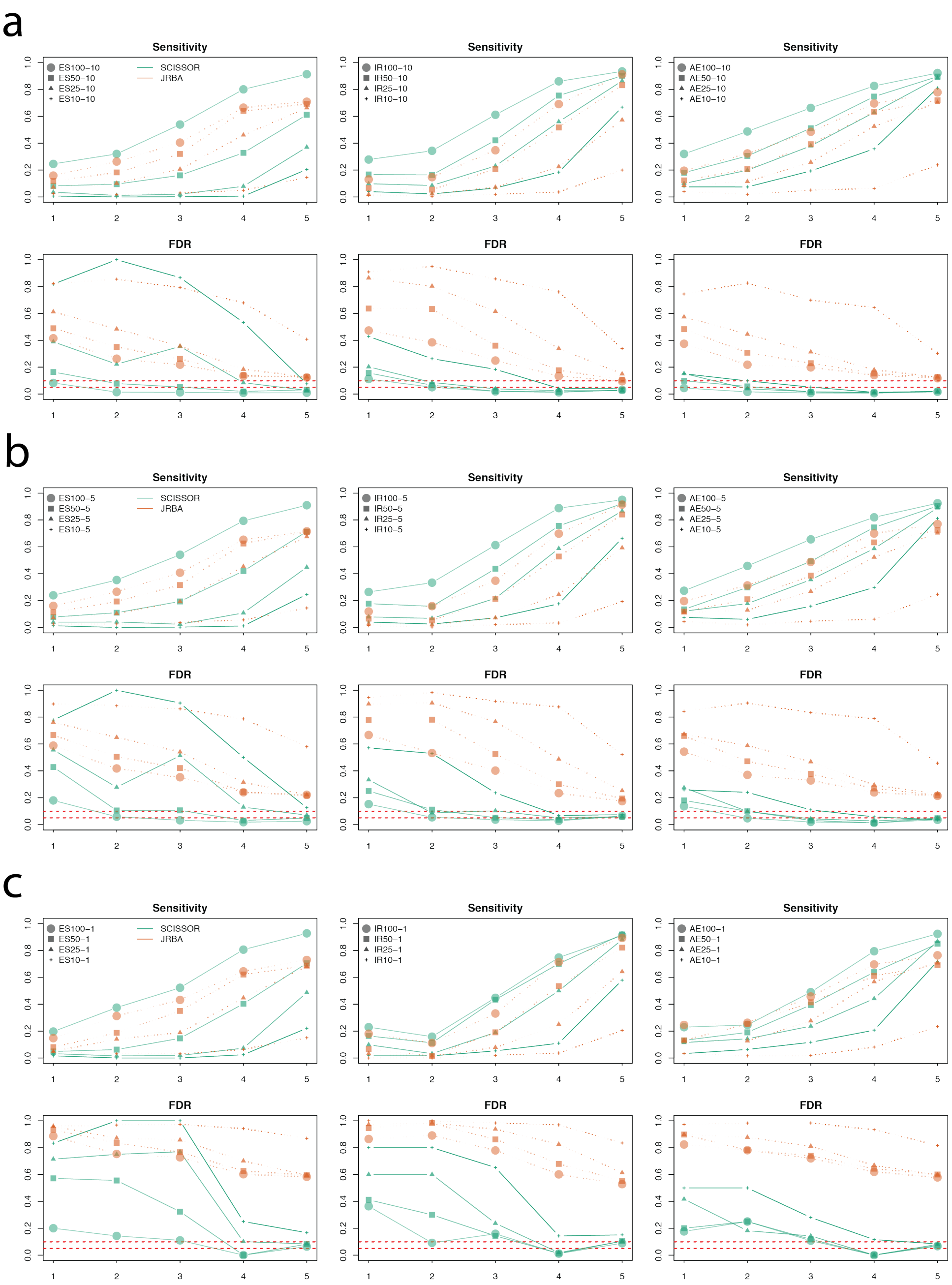

Supplementary Figure 20: Sensitivity and FDR for simulated RNA-seq data sets. In each panel, SCISSOR (orange) and JRBA (green) are compared according to gene expression levels (x-axis) and different shape change scenarios (left: exon skipping, middle: intron retention, right: alternative exon). (A) 5% outlier percentage (10 outliers) (B) 2.5% outlier percentage (5 outliers) (C) 0.5% outlier percentage (1 outlier)

Overall, both SCISSOR and JRBA showed higher sensitivities (true positive rates) in

genes expressed at higher levels than genes with lower expression (first row in each panel in Supplementary Figure 20). This is consistent to the results from real data analysis (Figure 3b) as changes in coverage are less noticeable if a gene is expressed at low levels. Note that both SCISSOR and JRBA did not show an excellent performance even for shape changes with 100% VAF at very high coverage levels. We found that many of the missing events (false negatives) were resulted by poor alignment in some proportion of the simulated genes. The junction reads in the impacted genes were not properly aligned, challenging both JRBA and SCISSOR by resulting in lack of junction reads and uneven coverage structure in their loci.

Except for a few cases, SCISSOR outperformed JRBA at every coverage level in the sensitivity as well as the FDR (second row of each panel in Supplementary Figure 20). Particularly, SCISSOR showed consistently low FDR (< 0.2 in most cases) whereas they were very high in JRBA. This simulation result is also consistent with the real data analysis (Figure 5) at the gene TP53. In Figure 5, we observed large numbers of false positives identified by JRBA particularly at low gene expression levels. This is mainly because of inevitable limitations from the ratio/percentage-based statistics which are difficult to determine outliers due to lack of the Gaussianity. On the other hand, the high-dimensional transformation offered by SCISSOR has the advantage that the resulting statistics are often roughly Gaussian, which enables accurate and robust identification of outliers. In addition to the normalization and skewness-adjusted outlier detection procedure by SCISSOR, linear transformations of high-dimensional data are theoretically known to be close to the Normal distribution under general assumptions as a result of the Central Limit Theorem.

Compared to the sensitivity plots presenting steadily increasing trends with respect to coverage levels, the FDR graphs showed some fluctuations. This is mostly due to the variation in the number of genes affected by poorly aligned junction reads as mentioned earlier. As more genes were affected, more false discoveries were observed, increasing FDR regardless of the coverage levels. As a result, JRBA has a number of false discoveries in those affected genes, substantially increasing its FDR. Nonetheless, SCISSOR achieved uniformly low FDR even under this circumstance, supporting the robustness of SCISSOR.

Considering different variant allele frequencies associated with shape changes, we see the

decreased sensitivities for the lower VAF in both SCISSOR and JRBA. This is expected because shape changes would become less apparent with lower VAF. For detecting retained introns and alternative exons, we were able to see that SCISSOR had higher sensitivities than JRBA in most VAF levels, and performs reasonably well even for outliers with low VAF such as 25% and 10%. For the exon skipping event, however, JRBA showed better sensitivities than SCISSOR for low VAF outliers. SCISSOR is most challenged when an exon is skipped with low VAF because the altered area becomes less variable after log-transformation. As we mentioned in the manuscript, this is one of the limitations of SCISSOR that would be addressed in a future study.

To examine the effect of outlier percentages on outlier detection accuracy, we considered 5%, 2.5%, and 0.5% (each panel of Supplementary Figure 20) of outliers in 200 samples. Sensitivity was not largely affected by outlier percentages in both methods. By contrast, FDR seemed to be significantly increased when only one outlier exists in data (0.5% case). This is because, by the definition of FDR (= FP/(TP+FP)), as the number of true outliers (TP) is low, the FDR will be dominated by false positives. Using the same significance level, therefore, it is the natural outcome that the lower outlier percentage has the larger FDR.

Supplementary Note 4:
Gene filtering method

In this section, we describe the angle-based measure for the level of shape-similarity (LSS) among samples.

For each gene,

1. Transform the base-level coverage counts, R_{ij} for base i and sample j, by $z_{ij} = \log_{10}(R_{ij} + 10) - \log_{10}(10)$. This shift parameter of 10 gave good experimental performance.

2. Define the 0-adjusted mean as $m_i^0 = \frac{1}{\sharp(z_{ij}>0)} \sum_{\{j:z_{ij}>0\}} z_{ij}$ for base i. We estimate the overall shape structure by the 0-adjusted mean vector, $m^0 = (m_1^0, \cdots, m_d^0)^T$.

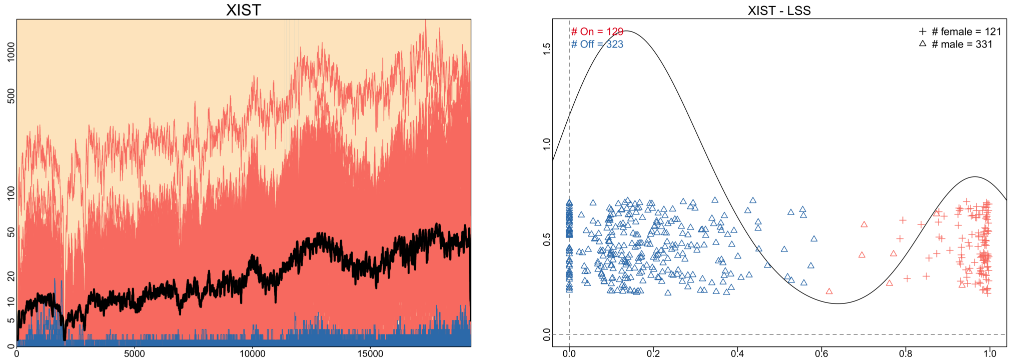

Supplementary Figure 21: On/Off analysis on *XIST*. The left plot shows the overlays of RNA-seq coverage with red color for on-samples and blue for off-samples classified from the on/off analysis. The right plot shows the estimated lss values with a kernel density estimate (black curve).Based on the cutoff value 0.6, the on- and off-samples were classified as indicated by red and blue colors. The gender of samples are also indicated by the triangle (△) for males and the cross symbol (+) for females. The on/off analysis clearly separates the data into two groups.

3. Compute the level of shape-similarity by $LSS_j = \frac{z_j^T m^0}{\|z_j\| \|m^0\|}$, which is equivalent to the cosine of the angle between the vectors Z_j and m^0 where $Z_j = (z_{1j}, \cdots, z_{dj})^T$.

4. Collect LSS_j $(j = 1, \cdots, n)$ for the given gene. If the percentage of samples with $LSS_j < 0.6$ is greater than 20%, we declare the gene to be an on/off gene.

For easier interpretation, we take the cosine transformation of the angles, which results in the values ranging from 0 to 1 with a higher value indicating closer to "on" (expressed) whereas a lower value indicates "off" (unexpressed). Then, the resulting values can be considered as the explained amounts of individual samples by the mean vector.

An on/off gene example is *XIST* shown in Supplementary Figure 21. This gene is associated with gender because it is involved in the X-chromosome inactivation. The left figure shows coverage of all samples at *XIST* with the 0-adjusted mean vector in black. The identified on/off samples using the proposed algorithm are represented by pink/blue colors. In contrast to the on-samples expressed at high or moderate levels, off-samples are rarely expressed for which the level and shape of gene expression is not statistically different from background noise. The LSS values used to separate on/off samples are shown on the left in Supplementary Figure 21 with the symbols +/△ for female/male. The two gender groups

are almost perfectly separated except for a few cases and this shows the strong association between gender and the on/off property at this gene.

Supplementary Note 5:
Modified projection outlyingness

As an analogue of a robust measure of outlyingness in 1-dimension, the projection outlyingness of y with respect to the data matrix Y for p-dimension is defined as $\max_{\|h\|=1} |\frac{h^T y - Med(h^T Y)}{MAD(h^T Y)}|$. The projection outlyingness finds the maximum outlyingness of a data point y by looking at all one-dimensional view of a dataset [6, 7, 9-12].

Here, we illustrate the projection outlyingness and the modified projection outlyingness using a toy data set. The toy data set was generated from the bivariate normal distribution with mean zero with one true outlier. Supplementary Figure 22 (a-d) illustrates the projection outlyingness of two data points in red and blue. Supplementary Figure 22 (a) shows the one-dimensional direction (red line) making the red point most outlying, meaning that the quantity $|\frac{h^T y - Med(h^T Y)}{MAD(h^T Y)}|$ is maximized when h is the red line. Supplementary Figure 22 (b) shows the projection scores obtained by projecting all the data points onto the red line. We can see that the red point is outlying in this one-dimensional representation and its projection score (≈ 6) is the projection outlyingness of the red point.

Similarly, Supplementary Figure 22 (c) shows the one-dimensional direction (blue line) that maximizes outlyingness of the blue point and Supplementary Figure 22 (d) shows the corresponding projection scores. Interestingly, the blue point has a larger projection outlyingness (> 6) than the red point. This illustrates the case when skewness can confound the projection outlyingness approach and thus may result in many false discoveries. The blue point is the data point farthest away from the center due to skewness of the distribution. In our study, such point is not considered as an outlier because its outlyingness comes from relatively strong involvement of some feature rather than its uniqueness.

It is often the case that RNA-seq coverage involve strong skewness, multi-modality, or high concentration near zero possibly due to existing clusters or a number of lowly expressed

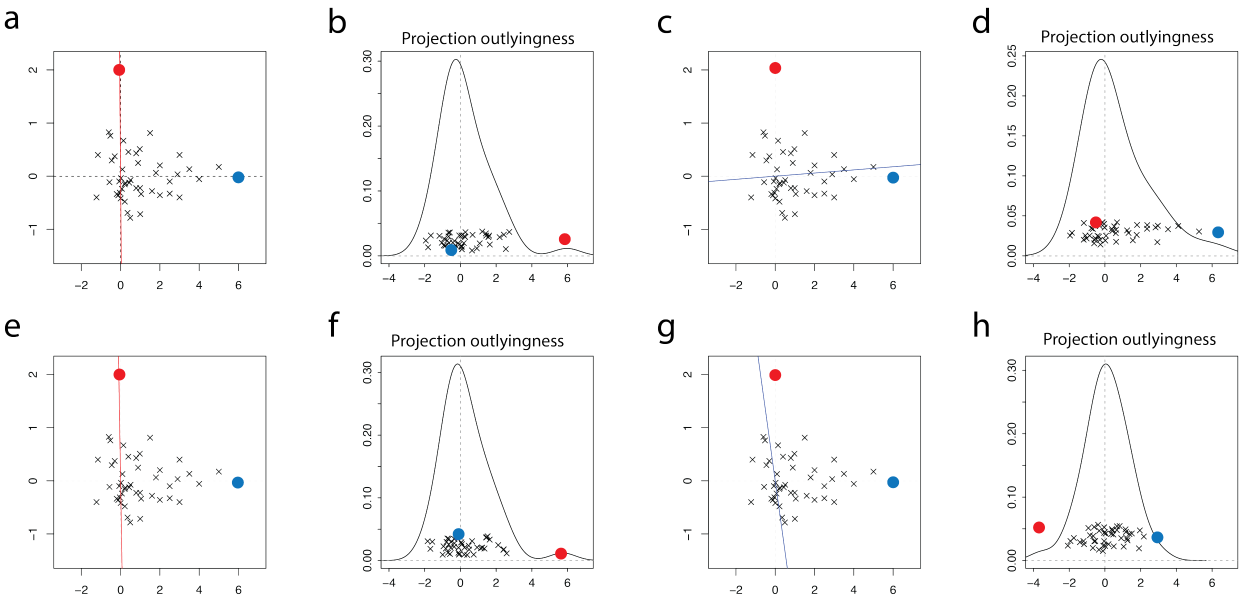

Supplementary Figure 22: A toy data set was generated from the bivariate normal distribution with mean zero. One outlier (red) and some skewed data points were added including the spuriously outlying point (blue). Top (a)-(d): the standard projection outlyingness approach was applied to this toy data set to identify outliers. The standard approach identified the blue point as the strongest outlier and the red point as the second strongest outlier. Bottom (e)-(h): the modified approach was applied to the same data set. The modified approach identified the red point as the only outlier and none of the spuriously outlying points were detected. (a) & (e): The red lines indicate the 2-dimensional directions, the most outlying directions (MODs), which maximize the outlyingness of the red point using the standard and the modified approach, respectively. (b) & (f): The standardized projection scores obtained by projecting all the data points onto the red lines in (a) & (e) are presented with the kernel density estimates. (c) & (g): The blue lines indicate the most outlying directions (MODs) that maximize the outlyingness of the blue point using the standard and the modified approach, respectively. (d) & (h) The standardized projection scores obtained by projecting all the data points onto the blue lines in (c) & (g) are shown with the kernel density estimates.

samples. Such departure from normality can produce false discoveries and conceal biologically important outliers as illustrated in Supplementary Figure 22 (c-d), and therefore some attention is needed. In the manuscript, we proposed a modified projection outlyingness approach taking into account departure from normality. To do this, we add normality constraint to the projection outlyingness function so that we only search the directions in which the given constraint is satisfied.

Supplementary Figure 22 (e-h) show the the results from our modified projection outly-

ingness approach. Supplementary Figure 22 (e,f) show the nearly same result as the standard projection outlyingness approach and the resulting statistic (projection outlyingness) for the red outlier is also similarly about 6. On the other hand, Supplementary Figure 22 (g,h) show the significantly improved result for the blue non-outlier. The modified approach does not search for the directions involved with severe skewness and therefore the resulting outlyingness of the blue point is now significantly decreased, which allows us to obtain reliable outlyingness statistics and avoid spurious outliers.

Supplementary Note 6:
Non-recurrent events in infrequently mutated genes

Supplementary Table 4 shows the distribution of the number of detected outliers across genes (n=14,399; on/off genes excluded) from the genome-wide analysis in the TCGA HNSCC cohort. About 32% of the genes had at least one significant shape changes, and many of them had only one or two shape changes identified. This shows the ability of SCISSOR to pick up non-recurrent outlier events. In addition, Supplementary Figure 23 shows the examples of non-recurrent shape changes identified in infrequently mutated genes where no recurrent truncating mutations were reported. In the gene *MAPK13*, we identified one sample involved with abnormally retained intron 11 (Supplementary Figure 23a). At this gene, only three missense mutations were reported in the TCGA HNSCC cohort with no truncating mutation. Another example is alternative transcript termination observed in a single sample for *LCP1* (Supplementary Figure 23b). This sample contains a novel *LCP1* isoform which contains the first seven exons of the gene and terminates in the middle of the following intron 7.

# of outliers (N)	$N = 0$	$N = 1$	$N = 2$	$N = 3$	$N = 4$	$N \geq 5$
# genes	9758	2523	1002	460	269	387
Percentage	67.8%	17.5%	7.0%	3.2%	1.9%	2.7%

Supplementary Table 4: Number of the identified outliers across genes.

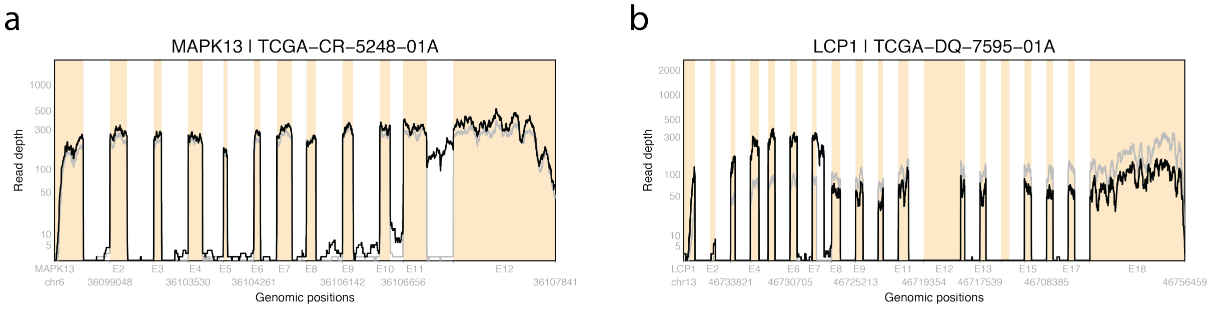

Supplementary Figure 23: Examples of non-recurrent shape changes identified in infrequently mutated genes where no recurrent truncating mutations were reported.

Supplementary Note 7:

Evaluation of small deletion events

To identify local shape changes in a limited region of base-level coverage such as small deletions, SCISSOR adopts sparse directions supporting biologically important regions as candidates where projection outlyingness would be considered. We consider the regions supported by abnormally spliced reads as well as sequential genomic regions by sliding a window along the locus. Using a collection of these sparse directions, we can accurately capture challenging local shape changes while reducing the impact of an overwhelming number of dimensions.

As with other windowing approaches, the selection of window sizes can impact the results. In this regard, we assessed SCISSOR with varying window sizes – 100, 75, 50, 10, 5, 1 – to systematically validate when base-level deletions are detectable by the method. The gene TP53 was used for the validation as it contains various sizes of deletions ($1 \sim 21$) in 50 samples (11%) of various expression levels. We considered three factors: deletion size, deleted read fraction, and gene expression. For each deletion event, we collected the deletion size which is the number of deleted nucleotides and deleted read fraction which is the fraction of reads missing the nucleotides. Normalized RSEM was used to measure gene expression of each sample.

At TP53, there were 34 deletions with length < 4, but they were mostly expressed at low levels or had low deleted read fraction, which did not substantially change their transcripts. To better understand the length limit that SCISSOR can identify, we deleted one nucleotide

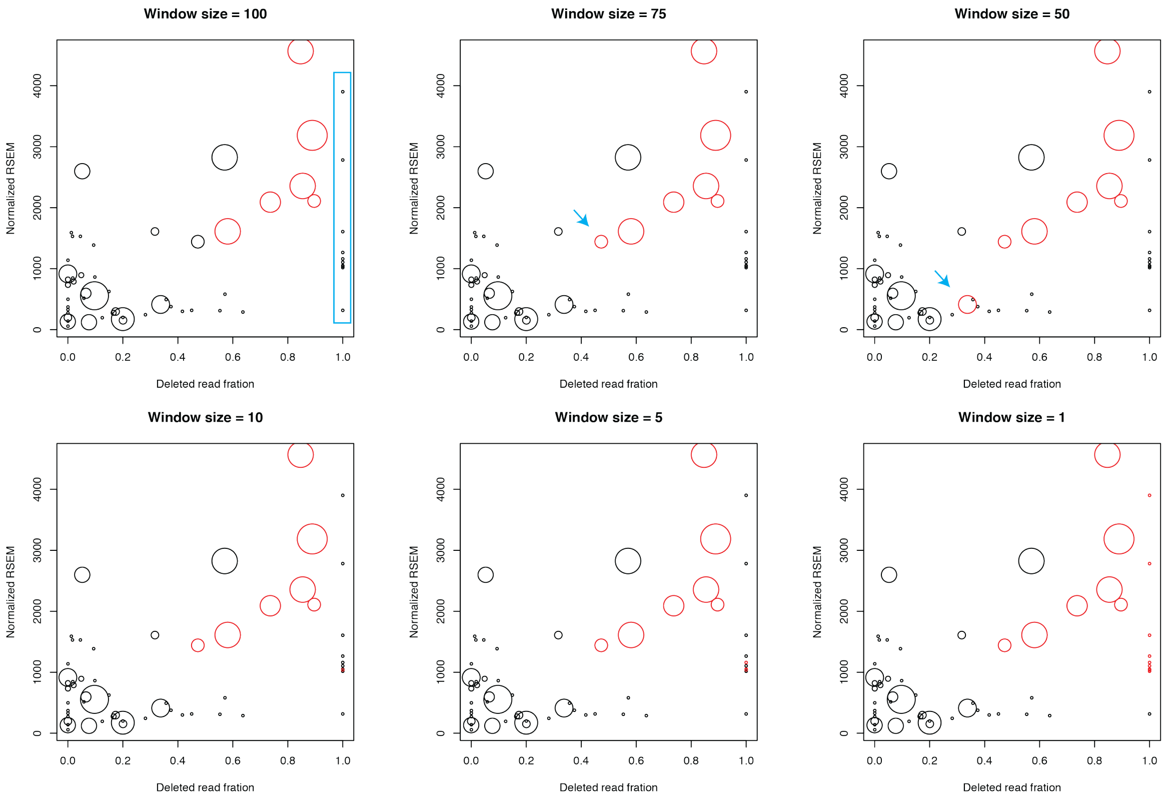

Supplementary Figure 24: Results of SCISSOR for detecting small deletion events (n=60) at gene TP53. In each panel, small deletion events are represented by circles with larger ones indicating larger lengths of deletions. The x-axis represents the fraction of reads missing the deleted nucleotides (deleted read fraction) and the y-axis represents the normalized RSEM. The identified deletions are highlighted by red. Among the 60 deletions, 10 one-nucleotide deletions at the right end of the x-axis where deleted read fraction=1 (the circles in the light blue rectangle in the top left panel) are the ones with randomly generated deletions. Some of the identified deletion events are specific to window sizes (e.g. circles indicated by arrows) and the one-nucleotide deletion events are identifiable with small window sizes.

in the read coverage of randomly chosen 10 samples at random locations. This added 10 more deletions with the 100% deleted read fraction at different expression levels as indicated by the light blue box in the top left panel in Supplementary Figure 24.

Supplementary Figure 24 shows the results for varying window sizes. In each panel, deletion events are represented with respect to the two axes - deleted read fraction and normalized RSEM with the larger circles for the larger deletions. The identified deletion events are indicated by red. With a given window size, SCISSOR tends to show better performance for larger deletion size, higher deleted read fraction, and higher gene expression. We were

able to observe that SCISSOR identified fairly small deletions, even nine nucleotides deleted with high outlyingness statistics. One deletion event (near at normalized RSEM=3000 and deleted read fraction=0.6) was not detected in any window size in spite of its large deletion size, medium level of deleted read fraction, and high gene expression. We found that this deletion event occurred nearby the splicing site where many other samples also have relatively lower read coverage so that the deletion event is challenging to detect relative to the low level of background coverage of this locus.

As we decreased the size of window, we were able to identify smaller deletions. Notably, SCISSOR with the window size=1 identified most of the one-nucleotide deletions except the case with very low expression as shown in the bottom right panel. Although the experiment only considered 100% read deletion, this clearly shows that SCISSOR has great potential for detecting deletions of the single nucleotide level. The disadvantage to decreasing window size is that the number of hypotheses tested is increased, thus lowering overall power and increasing the concern for false discoveries. Optimization of window size is an area for future development of the methods as mentioned in the discussion.

Supplementary Note 8: Evaluation of common structure of RNA-seq samples

To validate the assumption that common structure is shared by samples, we examined similarity of structure between technical and biological replicates. RNA was sequenced from total RNA of human bronchial epithelial (HBe) cells cultured in air-liquid interface (ALI) media at three differentiation stages (early: day3; intermediate: day 10; late: day 35). Specifically, HBe cells were collected from three healthy donors (DD030C, DD016F, DD046E) and histology was used to determine differentiation stage. Additionally, a HBe cell line, (UNCN3T) was cultured and sequenced in a similar manner. Each sample was repeated multiple times at the same condition as follows: 1× UNCN3T, 4× DD030C, 4× DD016F, 6× DD046E.

The replicates were compared to each other at randomly selected 1000 genes with high expression. Cosine similarity was used to measure how similar the replicates are in a high-

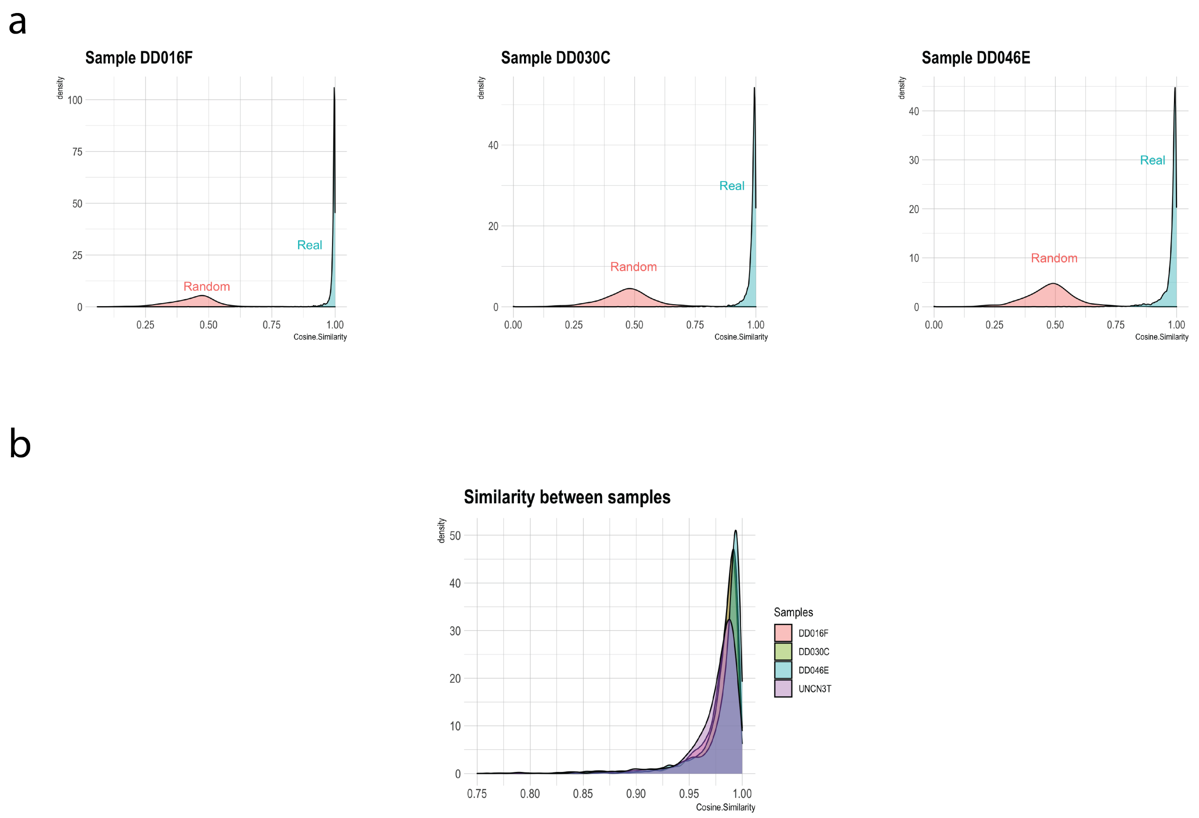

Supplementary Figure 25: Cosine similarity between technical replicates (a) and biological replicates (b)

dimensional space. Mathematically, cosine similarity is the cosine of the angle between two vectors, and thus cosine similarity$\approx$ 1 (angle$\approx$ 0°) indicates high similarity whereas cosine similarity$\approx$ 0 (angle$\approx$ 90°) indicates high discrepancy. Cosine similarity is advantageous particularly to our dataset because it does not depend on the size of vectors, which allows us to compare sample structures irrespective of their expression levels.

First, we investigated technical replicates using cosine similarity. Pairwise cosine similarities were measured within each sample at the randomly selected 1000 genes. The densities colored by cyan in Supplementary Figure 25a show very high similarity values within each sample, indicating that technical replicates exhibit highly similar coverage pattern.

To additionally confirm that the observed similarity values are significantly higher than the case with no common structure, we needed experimental similarity levels when two vectors did not have common structure so that we could compare the observed values with the experimental values. For this, we simulated a random noise replicate for every replicate,

which preserved the same expression level but in the absence of any structure. And then, pairwise cosine similarity was measured by replacing a replicate by its simulated replicate in every pair and the results are shown in the pink densities in Supplementary Figure 25a. In contrast to the real case, we were able to observe much lower similarity in the simulated replicates with no common structure, which additionally validates the common structure within sample.

Next, we further investigated the assumption for biological replicates (Supplementary Figure 25b). Having the observation that replicates within sample showed high similarity, the same idea can be applied to compare the replicates between samples. By taking one replicate of the sample DD016F as a fixed vector to compare (any replicate/sample can be chosen), we measured cosine similarity between the fixed vector and the other replicates from the four samples at the 1000 genes. Then, we obtained four groups of similarity values each of which corresponds to each sample. For example, the group corresponding to DD030C is a set of cosine similarity values between the fixed vector and the replicates from DD030C. We expect that the values from DD016F would be close to 1 because the fixed vector is its replicate. Using DD016F as a control, we can examine that the other samples also show the common structure shared with DD016F. As a result, Supplementary Figure 25b shows the density plots of the cosine similarity values from each of the four samples. The overlapping densities clearly demonstrate that the replicates from other samples also show high similarity with the fixed vector. Therefore, we can conclude that the four biological replicates have the common transcript structure, thus validating our underlying assumption.

References

[1] B. T. Sherman, R. A. Lempicki, *et al.*, "Systematic and integrative analysis of large gene lists using david bioinformatics resources," *Nature protocols*, vol. 4, no. 1, p. 44, 2009.

[2] C. G. A. Network *et al.*, "Comprehensive genomic characterization of head and neck squamous cell carcinomas," *Nature*, vol. 517, no. 7536, p. 576, 2015.

[3] S. Anders and W. Huber, "Differential expression analysis for sequence count data," *Genome biology*, vol. 11, no. 10, p. R106, 2010.

[4] M. D. Robinson and A. Oshlack, "A scaling normalization method for differential expression analysis of rna-seq data," *Genome biology*, vol. 11, no. 3, p. R25, 2010.

[5] J. H. Bullard, E. Purdom, K. D. Hansen, and S. Dudoit, "Evaluation of statistical methods for normalization and differential expression in mrna-seq experiments," *BMC bioinformatics*, vol. 11, no. 1, p. 94, 2010.

[6] B. Li and C. N. Dewey, "Rsem: accurate transcript quantification from rna-seq data with or without a reference genome," *BMC bioinformatics*, vol. 12, no. 1, p. 323, 2011.

[7] M.-A. Dillies, A. Rau, J. Aubert, C. Hennequet-Antier, M. Jeanmougin, N. Servant, C. Keime, G. Marot, D. Castel, J. Estelle, *et al.*, "A comprehensive evaluation of normalization methods for illumina high-throughput rna sequencing data analysis," *Briefings in bioinformatics*, vol. 14, no. 6, pp. 671–683, 2013.

[8] M. I. Love, W. Huber, and S. Anders, "Moderated estimation of fold change and dispersion for rna-seq data with deseq2," *Genome biology*, vol. 15, no. 12, p. 550, 2014.

[9] S. C. Hicks and R. A. Irizarry, "When to use quantile normalization?," *BioRxiv*, p. 012203, 2014.

[10] J. Zyprych-Walczak, A. Szabelska, L. Handschuh, K. Górczak, K. Klamecka, M. Figlerowicz, and I. Siatkowski, "The impact of normalization methods on rna-seq data analysis," *BioMed research international*, vol. 2015, 2015.

[11] R. Reddy, "A comparison of methods: normalizing high-throughput rna sequencing data," *bioRxiv*, p. 026062, 2015.

[12] P. K. Kimes, C. R. Cabanski, M. D. Wilkerson, N. Zhao, A. R. Johnson, C. M. Perou, L. Makowski, C. A. Maher, Y. Liu, J. S. Marron, *et al.*, "Sigfuge: single gene clustering of rna-seq reveals differential isoform usage among cancer samples," *Nucleic acids research*, vol. 42, no. 14, pp. e113–e113, 2014.

[13] H. Jung, D. Lee, J. Lee, D. Park, Y. J. Kim, W.-Y. Park, D. Hong, P. J. Park, and E. Lee, "Intron retention is a widespread mechanism of tumor-suppressor inactivation," *Nature genetics*, vol. 47, no. 11, p. 1242, 2015.

[14] Y. Song, O. B. Botvinnik, M. T. Lovci, B. Kakaradov, P. Liu, J. L. Xu, and G. W. Yeo, "Single-cell alternative splicing analysis with expedition reveals splicing dynamics during neuron differentiation," *Molecular cell*, vol. 67, no. 1, pp. 148–161. e5, 2017.

[15] Y. Katz, E. T. Wang, E. M. Airoldi, and C. B. Burge, "Analysis and design of rna sequencing experiments for identifying isoform regulation," *Nature methods*, vol. 7, no. 12, p. 1009, 2010.

[16] M. Seiler, S. Peng, A. A. Agrawal, J. Palacino, T. Teng, P. Zhu, P. G. Smith, S. J. Caesar-Johnson, J. A. Demchok, I. Felau, *et al.*, "Somatic mutational landscape of splicing factor genes and their functional consequences across 33 cancer types," *Cell reports*, vol. 23, no. 1, pp. 282–296, 2018.

[17] H. Climente-González, E. Porta-Pardo, A. Godzik, and E. Eyras, "The functional impact of alternative splicing in cancer," *Cell reports*, vol. 20, no. 9, pp. 2215–2226, 2017.

[18] E. Park, Z. Pan, Z. Zhang, L. Lin, and Y. Xing, "The expanding landscape of alternative splicing variation in human populations," *The American Journal of Human Genetics*, vol. 102, no. 1, pp. 11–26, 2018.

[19] A. C. Frazee, A. E. Jaffe, B. Langmead, and J. T. Leek, "Polyester: simulating rna-seq datasets with differential transcript expression," *Bioinformatics*, vol. 31, no. 17, pp. 2778–2784, 2015.
