# Supplementary material for: SCISSOR: a framework for identifying structural changes in RNA transcripts
Source: Nat Commun. 2021 Jan 12;12:286. doi: 10.1038/s41467-020-20593-3 (PMC7804101; doi:10.1038/s41467-020-20593-3)
Supplement: Supplementary file 3 — Reporting Summary [file 41467_2020_20593_MOESM3_ESM.pdf]

nature research

Corresponding author(s): D. Neil Hayes

Last updated by author(s): Nov 9, 2020

Reporting Summary

Nature Research wishes to improve the reproducibility of the work that we publish. This form provides structure for consistency and transparency in reporting. For further information on Nature Research policies, see Authors & Referees and the Editorial Policy Checklist.

Statistics

For all statistical analyses, confirm that the following items are present in the figure legend, table legend, main text, or Methods section.

n/a	Confirmed	
☐	☒	The exact sample size (*n*) for each experimental group/condition, given as a discrete number and unit of measurement
☒	☐	A statement on whether measurements were taken from distinct samples or whether the same sample was measured repeatedly
☐	☒	The statistical test(s) used AND whether they are one- or two-sided<br>*Only common tests should be described solely by name; describe more complex techniques in the Methods section.*
☒	☐	A description of all covariates tested
☐	☒	A description of any assumptions or corrections, such as tests of normality and adjustment for multiple comparisons
☐	☒	A full description of the statistical parameters including central tendency (e.g. means) or other basic estimates (e.g. regression coefficient) AND variation (e.g. standard deviation) or associated estimates of uncertainty (e.g. confidence intervals)
☐	☒	For null hypothesis testing, the test statistic (e.g. *F*, *t*, *r*) with confidence intervals, effect sizes, degrees of freedom and *P* value noted<br>*Give P values as exact values whenever suitable.*
☒	☐	For Bayesian analysis, information on the choice of priors and Markov chain Monte Carlo settings
☒	☐	For hierarchical and complex designs, identification of the appropriate level for tests and full reporting of outcomes
☒	☐	Estimates of effect sizes (e.g. Cohen's *d*, Pearson's *r*), indicating how they were calculated

Our web collection on statistics for biologists contains articles on many of the points above.

Software and code

Policy information about availability of computer code

Data collection	samtools 1.9, R 3.5.3, TopHat 2.0.14, and python 2.7.14 were used in this study
Data analysis	The analysis tool being presented here is written for R, version 3.4 and higher, and is available at www.github.com/hyochoi/SCISSOR.

For manuscripts utilizing custom algorithms or software that are central to the research but not yet described in published literature, software must be made available to editors/reviewers. We strongly encourage code deposition in a community repository (e.g. GitHub). See the Nature Research guidelines for submitting code & software for further information.

Data

Policy information about availability of data

All manuscripts must include a data availability statement. This statement should provide the following information, where applicable:

- Accession codes, unique identifiers, or web links for publicly available datasets
- A list of figures that have associated raw data
- A description of any restrictions on data availability

All sequencing data as binary alignment (BAM) format for TCGA head and neck squamous cell carcinoma cancer patients were downloaded from the TCGA Data Portal (https://portal.gdc.cancer.gov/). The mutation data were downloaded at https://gdac.broadinstitute.org/. The gene annotation file was downloaded at https://gdc.cancer.gov/about-data/data-harmonization-and-generation/gdc-reference-files.

Field-specific reporting

Please select the one below that is the best fit for your research. If you are not sure, read the appropriate sections before making your selection.

☒ Life sciences ☐ Behavioural & social sciences ☐ Ecological, evolutionary & environmental sciences

For a reference copy of the document with all sections, see nature.com/documents/nr-reporting-summary-flat.pdf

Life sciences study design

All studies must disclose on these points even when the disclosure is negative.

Sample size	Sample size was determined by the number of cases available in the TCGA database. RNA-seq data for HNSC were available for 522 tumor specimens and 44 normal specimens.
Data exclusions	No sample was excluded from the analysis.
Replication	In the validation of common structure shared by RNA-seq samples, RNA was sequenced from total RNA of human bronchial epithelial (HBe) cells cultured in air-liquid interface (ALI) media at three differentiation stages (early: day3; intermediate: day 10; late: day 35). Specifically, HBe cells were collected from three healthy donors and histology was used to determine differentiation stage. Additionally, a HBe cell line was cultured and sequenced in a similar manner. Each sample was repeated multiple times at the same condition.
Randomization	Bootstrapping with the TCGA HNSC samples was used for computing error bands for the prediction of known variants. For the rest of the study, all available samples from the TCGA HNSC were included.
Blinding	No blinding was performed in this study. Blinding was not possible as the study was unsupervised and all samples had to be analyzed and compared together for the goal of detecting the ones that were significantly different from the majority of samples.

Reporting for specific materials, systems and methods

We require information from authors about some types of materials, experimental systems and methods used in many studies. Here, indicate whether each material, system or method listed is relevant to your study. If you are not sure if a list item applies to your research, read the appropriate section before selecting a response.

Materials & experimental systems

n/a	Involved in the study	
☒	☐	Antibodies
☒	☐	Eukaryotic cell lines
☒	☐	Palaeontology
☒	☐	Animals and other organisms
☒	☐	Human research participants
☒	☐	Clinical data

Methods

n/a	Involved in the study	
☒	☐	ChIP-seq
☒	☐	Flow cytometry
☒	☐	MRI-based neuroimaging
